# Integrative genomic mining for enzyme function to enable engineering of a non-natural biosynthetic pathway

**DOI:** 10.1038/ncomms10005

**Published:** 2015-11-24

**Authors:** Wai Shun Mak, Stephen Tran, Ryan Marcheschi, Steve Bertolani, James Thompson, David Baker, James C. Liao, Justin B. Siegel

**Affiliations:** 1 Department of Chemistry, University of California Davis, One Shields Avenue, Davis, California 95616, USA; 2 Department of Bioinformatics, University of California, Los Angeles, 172 Boyer Hall Box 951570, 611 Charles E. Young Drive East, Los Angeles, California 90095-1570, USA; 3 Department of Chemical and Biomolecular Engineering, University of California Los Angeles, 5531 Boelter Hall, Los Angeles, California 90095-1592, USA; 4 Department of Biochemistry, University of Washington, 1705 NE Pacific St Seattle, Washington 98195-7350, USA; 5 Institute for Genomics and Proteomics, University of California Los Angeles, 611 Young Drive East, Los Angeles, California 90095, USA; 6 Department of Biochemistry and Molecular Medicine, University of California Davis, 2700 Stockton Boulevard, Suite 2102, Sacramento, California 95817, USA; 7 Genome Center, University of California Davis, 451 Health Sciences Drive, Davis, California 95616, USA

## Abstract

The ability to biosynthetically produce chemicals beyond what is commonly found in Nature requires the discovery of novel enzyme function. Here we utilize two approaches to discover enzymes that enable specific production of longer-chain (C_5_–C_8_) alcohols from sugar. The first approach combines bioinformatics and molecular modelling to mine sequence databases, resulting in a diverse panel of enzymes capable of catalysing the targeted reaction. The median catalytic efficiency of the computationally selected enzymes is 75-fold greater than a panel of naively selected homologues. This integrative genomic mining approach establishes a unique avenue for enzyme function discovery in the rapidly expanding sequence databases. The second approach uses computational enzyme design to reprogramme specificity. Both approaches result in enzymes with >100-fold increase in specificity for the targeted reaction. When enzymes from either approach are integrated *in vivo*, longer-chain alcohol production increases over 10-fold and represents >95% of the total alcohol products.

## Abstract (web summary)

The modification of enzymes to generate desired compounds by non-natural pathways is a useful route for the production of commodity chemicals. Here, the authors show two approaches—genome mining and computational enzyme design—to generate higher alcohols from sugar.

## Graphical abstract

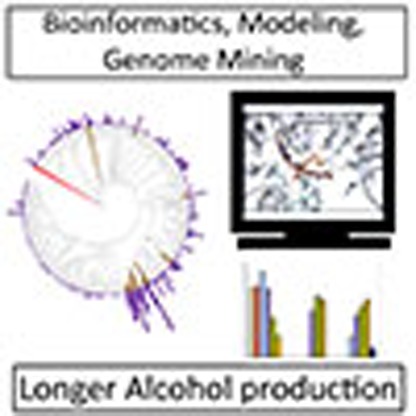

A central goal of the bioeconomy is to reduce our dependence on petroleum through next-generation biomanufacturing^1^. The USDA projects the industrial production of bio-based specialty chemicals to reach ∼$340 billion USD by 2025, replacing half of our needs for these products from petroleum^2^. To accomplish this ambitious goal, non-natural biochemical pathways are needed. An example of one such a pathway is the ‘synthetic recursive +1' carbon elongation pathway that has been developed to produce a variety of alcohol products in *Escherichia coli*^3^,^4^. The primary product from the current pathway is 1-butanol, with longer-chain alcohols (for example, pentanol, hexanol, heptanol and octanol) being either minor products of the pathway or not produced at all^3^,^4^. Yet, there is significant interest in producing longer-chain alcohols, given their use as specialty chemicals^5^, as well as their greater energy density when used as liquid fuels or fuel additives. To produce longer-chain alcohols through the synthetic recursive +1 carbon elongation pathway, we demonstrate that modulating the specificity of a single enzyme in the pathway could redirect metabolic flux towards longer-chain alcohols.

In the current synthetic recursive +1 pathway, the enzymes LeuA, LeuB, LeuC and LeuD (collectively known as LeuABCD) are recruited to recursively elongate 2-ketobutyrate into long-chain 2-ketoacids^3^,^4^. These 2-ketoacids are then converted to aldehydes by ketoisovalerate decarboxylase^6^ (KIVD) and subsequently to alcohols by alcohol dehydrogenase^3^ (ADH; Fig. 1). In this engineered pathway, the primary determinant for the final product chain length is the number of cycles through LeuABCD^7^.

Previous efforts to engineer the product specificity of this pathway have focused on broadening the specificity of LeuA and KIVD^3^. When the specificity of LeuA is broadened, additional cycles through the pathway are carried out, resulting in a mixture of C_2_–C_6_ linear and branched alcohols^3^. When the specificity of both LeuA and KIVD are broadened, C_2_–C_8_ products are produced with major products being butanol and pentanol^4^. To enhance longer-chain alcohol production, we focused on discovering a ketoacid decarboxylase specific for long-chain ketoacids. By replacing KIVD with an enzyme specific for long-chain ketoacids, the short-chain substrates were able to re-enter the +1 iteration cycle until their conversion to long-chain ketoacids, which are then decarboxylated and reduced into longer chain alcohols.

We employed two computationally directed approaches to discover a decarboxylase with the desired functional profile. The first is a new approach to mine the rapidly growing sequence databases. The majority of these proteins derived from the genomic sequencing of organisms (that is, genomic enzyme orthologues: GEOs) have not been experimentally characterized. Therefore, neither function nor specificity is known. Previous efforts to mine GEOs for function have relied on random sampling or sequence-based bioinformatics over an entire enzyme family^8^,^9^,^10^. These methods require a large number of genes to be produced and experimentally characterized. To enable a more efficient sampling method, we developed a novel computational pipeline that integrates molecular modelling and bioinformatics to forward predict GEOs' ability on carrying out the function of interest. Using this integrative genomic mining approach we identify a set of ketoacid decarboxylases diverse in sequence, but capable of utilizing 2-ketooctanoate (C8) as a substrate. In addition, we demonstrate that the median activity of GEOs selected using the integrative genomic mining approach is 75-fold greater than a set of naively selected proteins from the enzyme family. The second approach focuses on reprogramming the substrate specificity of KIVD by using computational enzyme design methods. Using the Rosetta Molecular Modeling Suite to direct the generation and screening of over 400 mutants results in the discovery of a mutant with >600-fold increase in specificity for C8 (ref. 11). Finally, the most specific and active enzyme from each approach is incorporated into the synthetic recursive +1 pathway to evaluate their ability to modulate *in vivo* alcohol production. Both enzymes result in a complete switch of the alcohol production profile towards longer chain alcohols, demonstrating the ability to rationally control biosynthetic product specificity for chemicals not commonly found in Nature.

## Results

### Integrative genomic mining for enzyme function

**Figure 2:**
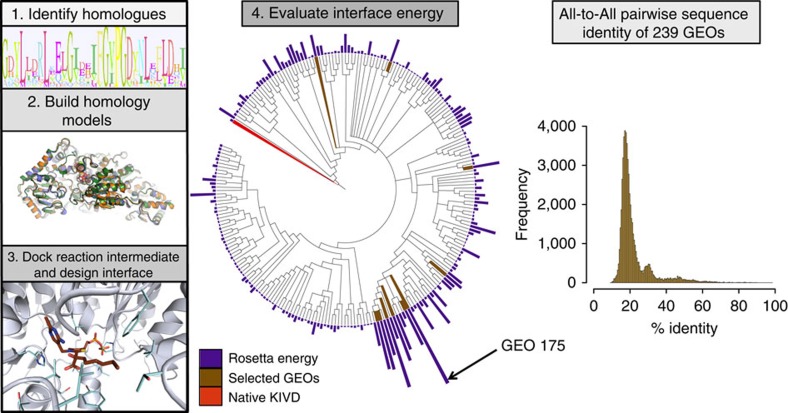
The computationally directed genomic enzyme-mining pipeline. GEOs were identified based on amino-acid sequence homology to KIVD. Bioinformatics filters were used to identify a set of amino-acid sequences from genomic databases that are predicted to be KIVD homologues and likely to be decarboxylases. Once identified, homology models were built to obtain a predicted ternary structure of each GEO. Ligand docking and design simulations were subsequently run in the presence of our target ligand (for example, C8) to evaluate the potential protein–ligand interface energy. A phylogenetic tree for the 239 GEOs is depicted with a bar chart above each sequence. The bar height indicates the predicted protein–ligand interface energy; the higher the bar the lower the energy. Bar height is scaled linearly relative to the lowest protein–ligand interface energy. Ten GEOs (brown) were selected for experimental characterization. The pairwise sequence identity of all 239 GEOs to each other has a mode of 20%, indicating the high level of sequence diversity within this set of sequences.

KIVD belongs to the TPP enzyme family that is composed of more than 17,000 sequences^12^. To identify GEOs with the desired function within this vast sequence space, we combined bioinformatics^13^ and molecular modelling^14^ with functional constraints to guide our exploration (Fig. 2). First, ketoacid decarboxylase GEOs were identified based on sequence similarity to the KIVD used in the current synthetic recursive +1 pathway. At the time the search was performed, 2,082 sequences were found in the non-redundant sequence database as significant matches. To broadly search KIVD sequence space, redundant sequences were filtered out with a sequence identity cutoff of 90% (ref. 15). Sequences derived from eukaryotic organisms were removed to increase the likelihood of producing soluble proteins in *E. coli* where the synthetic recursive +1 pathway has been implemented.

We assumed that all potential scaffolds with the desired function should be structurally homologous to KIVD, and further refined our sequence set accordingly. Since crystal structures for most of these predicted proteins are not available for analysis, we produced homology models to obtain a predicted ternary structure. Using Rosetta Comparative Modeling, one hundred models were generated for each of the 239 GEOs^14^. The lowest-energy model was selected as the representative for each GEO and evaluated for structural similarity to KIVD. We used the TMalign algorithm^16^ to overlay the models with the native KIVD crystal structure, and only those with a TMalign score of >0.5 were kept. These bioinformatics and structural filters result in 239 GEOs as candidates for the targeted function (Supplementary Table 1). The pairwise identity of every sequence to each other was calculated and the mode of these values lies near 20% (Fig. 2). Thus, the selected set of 239 GEOs represents a diverse sampling of sequence space for this fold family.

To further enrich the set for GEOs likely to function on C8, we docked a modelled reaction intermediate of the C8 substrate within the predicted active site (Fig. 2). The lowest-energy model for each GEO was used as a template for docking a modelled C8 intermediate with functional constraints, which ensured a productive geometric orientation between the predicted catalytic residues in the protein and intermediate. The C8 reaction intermediate used for docking is based on the synthetic thiamine pyrophosphate (TPP) analogue from a crystal structure of KIVD (PDB ID: 2VBG), which mimics the initial nucleophilic attack by the thiazolium^17^. Docking calculations were carried out using Rosetta Design in which both the identity and conformation of non-catalytic residues within the active site were allowed to change, and the backbone was allowed to move during minimization. Although the C8 intermediate may be docked without the introduction of mutations, the design was enabled during the simulations to allow for the possibility of introducing conservative mutations that significantly reduce interface energy. After docking and design simulations, the calculated interface energy was used to evaluate each GEO's ability to accommodate C8.

A phylogenetic tree based on sequence similarity was built for the 239 GEOs in which the lowest protein–ligand interface energy from the Rosetta Design simulation for each GEO are depicted (Fig. 2). The bar height above each GEO leaf is proportional to the lowest interface energy from all 239 GEOs, the higher the bar the lower the energy. On the basis of the calculated energies it is immediately apparent that a cluster of GEOs distant in sequence to KIVD (∼15% sequence identity) is predicted to be capable of utilizing C8. The enzyme with the lowest energy, GEO 175, is a predicted protein in the genome database with no known function. The enzyme with the closest amino-acid sequence identity to GEO 175 (∼50% sequence identity) for which significant biophysical characterization has been performed is benzoylformate decarboxylase from bacteria *Pseudomonas putida* (ppBFD)^18^. A structural analysis of the active site of ppBFD and GEO 175 reveals that the active sites are only 50% identical in sequence (Supplementary Fig. 1), and the activity of ppBFD on C8 has not been previously studied.

**Figure 3:**
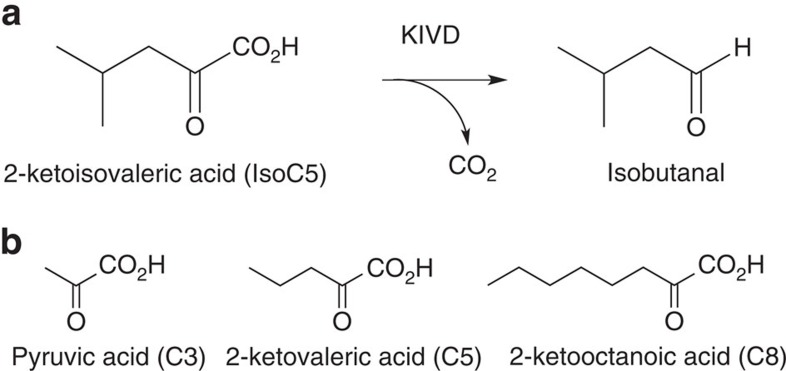
Characterized reaction specificities. (**a**) KIVD utilizes various ketoacids as substrates to produce the corresponding aldehyde. (**b**) Ketoacids used for *in vitro* kinetic constant characterization in addition to 2-ketoisovaleric acid.

From the design simulations, 10 GEOs were chosen for experimental characterization based on the predicted protein–ligand interface energy as well as sequence diversity (Fig. 2 and Supplementary Table 1). Before ordering these GEOs, each mutation introduced through the Rosetta Design simulations was manually analysed. Mutations introduced during the design simulations were reverted if they were not found to significantly enhance the calculated Rosetta interface energy. Synthetic genes encoding each enzyme was obtained, expressed in *E. coli* and purified for *in vitro* kinetic characterization using a panel of 2-ketoacid substrates of different chain lengths including C8, C5, C3 and the natural substrate for KIVD, ketoisovaleric acid (Fig. 3). Out of the 10 selected GEOs, 6 (GEO 41, 74, 110, 175, 179 and 195) express and are able to be purified in a soluble form using our standardized method. Of these six, three exhibit detectable activities on at least one of the four ketoacids used for kinetic characterization.

**Figure 4:**
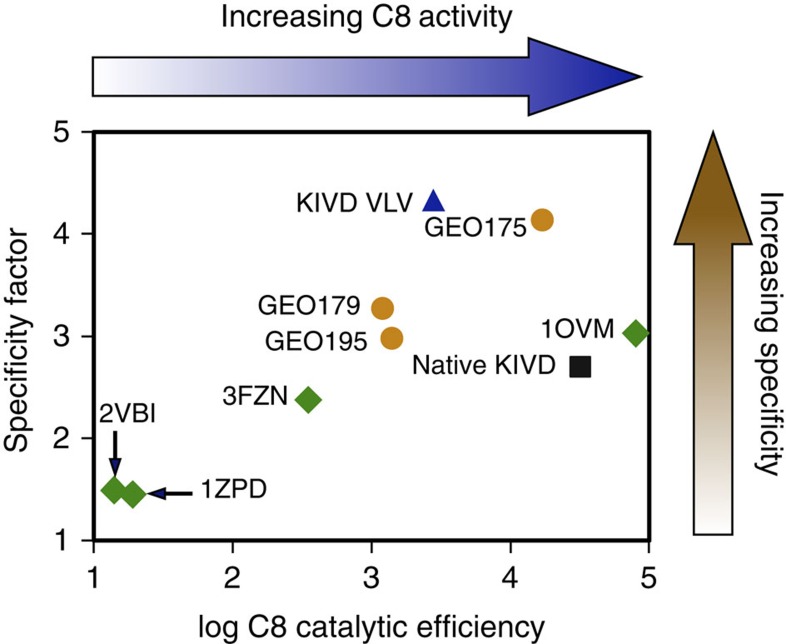
Catalytic efficiency and specificity of characterized ketoacid decarboxylases. Specificity factor is calculated as 
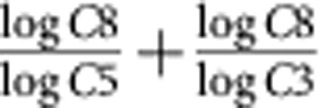
 where the log-scaled catalytic efficiencies for C8 relative to C5 and C3 are compared. The three active GEOs and the naively selected set of decarboxylases are represented in gold (circles) and green (diamonds), respectively. Native KIVD and KIVD_VLV are depicted in black (square) and blue (triangle), respectively. 1OZF is not included in this figure since activity on C8 is below our limit of detection. The genomic mining method and computational active-site redesign approach both produced enzymes with enhanced specificity relative to native KIVD.

Among the active GEOs, the enzyme with the highest efficiency on C8 is the computationally top ranked GEO 175, with a *k*_cat_/*K*_M_ of 17,000 M^−1^s^−1^ (Table 1 and Fig. 4). This is only twofold lower than the efficiency of native KIVD on C8. However, GEO 175's catalytic efficiency on C8 is 33,000-fold higher than that on C3 and 354-fold higher than that on C5. When assayed under the same conditions, native KIVD's catalytic efficiency on C8 is 762-fold higher than that on C3 and 3.4-fold higher than that on C5. This corresponds to over a 100-fold improvement in specificity (C8 versus C5) relative to the native KIVD.

To evaluate whether the integrative genomic mining approach enriches the mining for C8 activity, an additional set of previously characterized ketoacid decarboxylases diverse in sequence were experimentally characterized in an equivalent manner. Five enzymes were selected (PDB: 1OVM, 2VBI, 3FZN, 1ZPD and 1OZF) and all were found to have detectable activity on at least one of the four ketoacids used for GEO characterization. The median C8 activity from the GEOs selected using the integrative genomic mining approach is 75-fold higher than the naively picked decarboxylases (Table 1 and Fig. 4). This indicates that the integrative genomic mining approach is effective in identifying functional enzymes that perform the desired reaction on C8.

**Figure 5:**
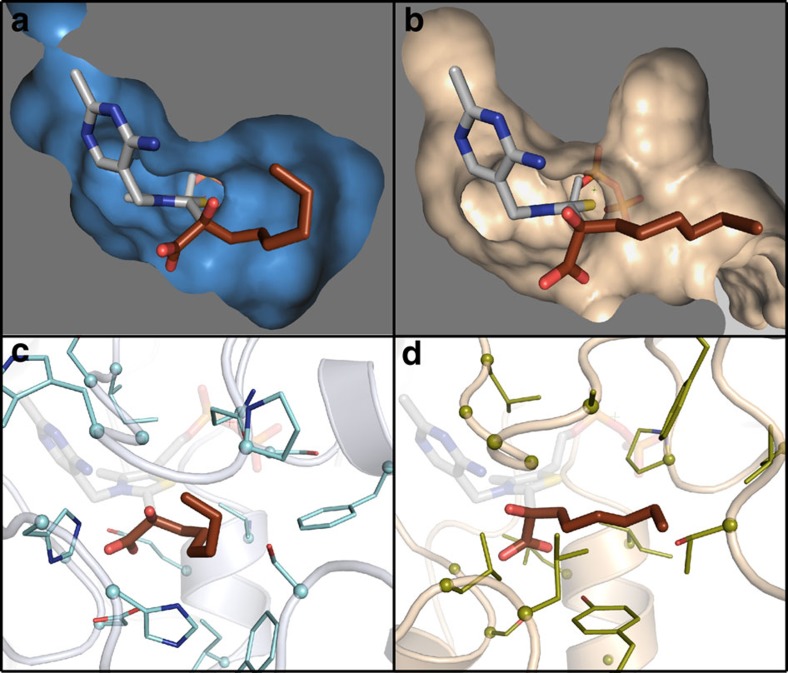
Molecular model of the C8 intermediate docked into KIVD and GEO 175. Cross-sections of the space-filled active site are represented for KIVD (**a**) and GEO 175 (**b**). The C8 ketoacid and TPP ligand are shown in brown and grey, respectively. The relative orientation of the C8 ligand to the TPP cofactor represents one conformation of the reaction intermediate used in the simulations; additional details are provided in Methods. A detailed view of the amino acids within the active site are illustrated for KIVD (**c**) and GEO 175 (**d**). Residues within 5 Å of C8 ligand are shown in sticks and their corresponding C-alpha are shown in spheres. Figure was generated using PyMol v1.7.4 (ref. 34).

**Figure 6:**
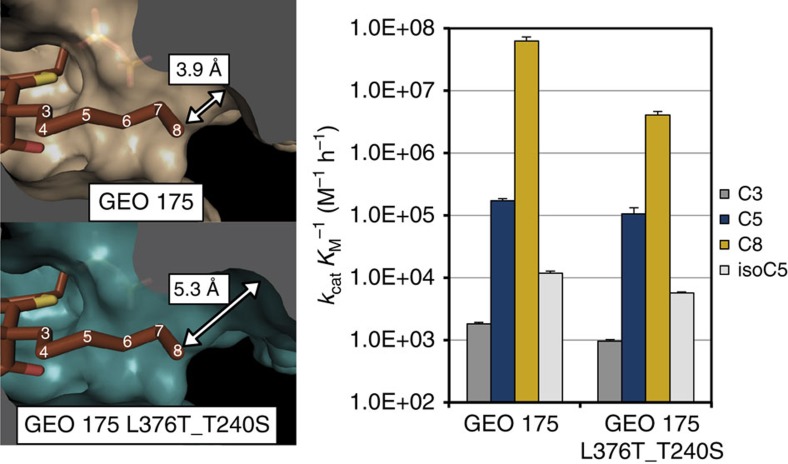
Kinetic characterization of GEO 175 and GEO 175 L376T_T240S. Kinetic constants were measured as described in Methods. The substrate 2-ketooctanoate carbon chain numbering referred to in the text is numbered in white. According to the molecular model of GEO 175, the double mutation L376T_T240S is predicted to recede the pocket by 1.4 Å and remove interactions between the binding pocket and carbon 8 of the ketoacid alkyl chain. This mutant was observed to decrease catalytic efficiency on C8, but has a negligible effect on shorter-chain substrates. Errors are shown as ±one s.d.

The amino-acid sequence for GEO 175 is not predicted to require any mutations to be capable of utilizing the C8 substrate, and therefore represents the native enzyme from *Streptomyces sp. C*. Comparing the active-site pocket of GEO 175 with native KIVD (16.8% identity to each other) reveals significant shape differences (Fig. 5). On the basis of molecular modelling, GEO 175 is predicted to have an extended active site that is significantly more open to solvent relative to native KIVD. This results in the predicted binding mode for C8 to be extended in GEO 175 as opposed to a ‘wrapped' conformation in KIVD. In GEO175, the third through fifth carbons of the C8 ketoacid ligand are predicted to occupy a solvent-exposed, open pocket with limited molecular interactions. However, the sixth through eighth carbons are predicted to interact with a narrow and hydrophobic pocket. Contrary to GEO 175, KIVD has an enclosed hydrophobic binding pocket and is predicted to make molecular interactions throughout the ketoacid alkyl chain (Figs 5 and 6).

On the basis of these observations we engineered a mutant of GEO 175 containing two amino-acid changes (L376T and T240S) predicted to remove the direct molecular interactions with seventh and eighth carbons of the ketoacid alkyl chain, while maintaining overall protein stability. On kinetic characterization, we found that the mutant protein's catalytic efficiency on C8 is 15-fold lower than GEO 175, but the decrease in efficiency for C5, C3 and isoC5 assayed under the same conditions is 2-fold or less (Table 1 and Fig. 6). The ability to rationally modulate activity based on structure supports the accuracy of the GEO 175 molecular model and the hypothesis that the specificity of GEO 175 can be attributed to a combination of hydrophobic interactions with the sixth through eighth carbons of the alkyl chain, while lacking interactions with the third through fifth carbons (Fig. 6).

### Computationally directed KIVD-active-site redesign

**Figure 7:**
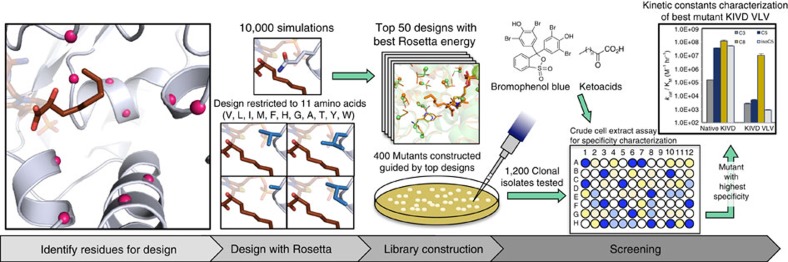
Screening process for identifying an engineered KIVD with altered substrate specificity. The design process started with identifying active-site residues of KIVD (depicted in red). These amino acids were allowed to either remain native or sample any of 11 relatively hydrophobic amino acids. A total of 10,000 design simulations were run and the amino acids identified in the 50 lowest-energy designs were used to guide construction of a small library of roughly 400 KIVD mutants from the original combinatorial space of 10^11^ possible active-site mutations. Overall, 1,200 clonal isolates were screened for activity and specificity. The KIVD_VLV mutant was selected and subsequently purified for *in vitro* kinetic constant characterization.

In our second approach to improve the specificity of KIVD, we used Rosetta Design methods to explore the potential active-site sequence space that would accommodate 2-ketooctanoate as a substrate^11^. Design simulations were carried out with the same reaction intermediate and functional constraints utilized in the genomic mining pipeline. Ten residues in the active site were targeted for mutagenesis. At each site, one of eleven relatively hydrophobic amino acids (Val, Leu, Ile, Met, Phe, His, Gly, Ala, Thr, Tyr and Trp) was systematically introduced, and the remaining nine sites were allowed to be redesigned. The identities of amino acids at all other residues were kept constant (Fig. 7). The sequence profile from the 50 lowest-energy designs was used to guide the construction of a small library of roughly 400 KIVD mutants from the original combinatorial space of over 10^11^ possible active-site mutations.

Since KIVD can already efficiently utilize C8 as a substrate, enzyme specificity is of primary importance. Therefore, each mutant was screened against a panel of 2-ketoacid substrates to assess the specificity and activity of each mutant (Supplementary Fig. 2). This assay was performed using a high-throughput pH-dependent colorimetric assay of enzyme activity in crude cell extracts. Mutations that increased specificity for C8 were then combined to produce combinatorial mutants and screened for specificity with the colorimetric assays. The mutant that exhibits the highest specificity and activity is G402V/M538L/F542V (KIVD_VLV). Kinetic constant characterization on purified enzymes shows that the engineered enzyme KIVD_VLV has a 600-fold improvement in specificity between C8 and C5, in terms of catalytic efficiency, relative to the native KIVD (Table 1).

### *In vivo* testing of longer-chain alcohol production

The measured specificity and activity for each enzyme was used to select two candidates for further characterization of their ability to modulate alcohol production *in vivo*. The two enzymes with the highest activity and specificity (Fig. 4), KIVD_VLV and GEO 175, were tested for their ability to reprogramme the synthetic recursive +1 pathway to produce longer-alcohol products *in vivo*. The alcohol-producing biochemical pathway was adapted from a previous study used to synthesize alcohols of various lengths^3^,^4^. Starting from glucose, the aforementioned +1 pathway, involving LeuABCD, recursively elongates 2-ketoacids, starting with 2-ketobutyrate, into long-chained 2-ketoacids. These 2-ketoacids are then converted to aldehydes by ketoacid decarboxylases (native KIVD, KIVD_VLV and GEO 175) and subsequently to alcohols by an ADH. The carbon flux through this alcohol-synthesizing biochemical pathway is enhanced by overexpressing thrABC, ilvA, LeuABCD and ADH6 on inducible plasmids transformed into *E. coli*^3^. The effects of overexpression were, furthermore, supported and maintained by knocking out the threonine exporter enzyme RhtA and the primary DNA recombination degradation enzyme RecA. The enzyme AdhE was also knocked out, which helps raise longer-chain alcohol production by eliminating a competing pathway where acetyl-CoA is directly converted to ethanol^19^.

**Figure 8:**
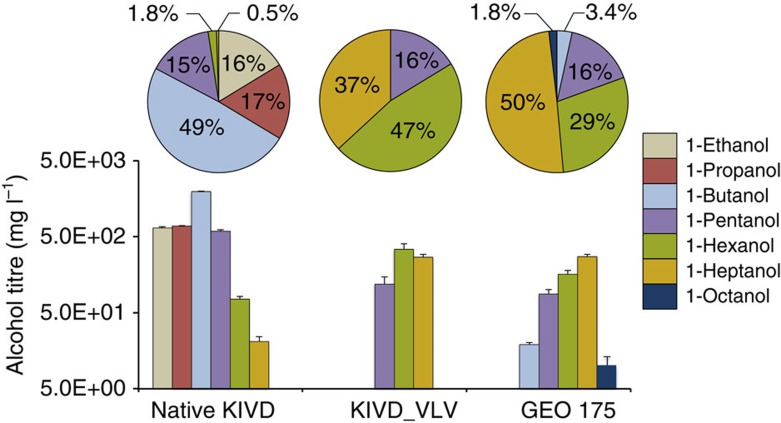
*In vivo* alcohol production of the synthetic recursive +1 pathway with native KIVD, KIVD_VLV and GEO 175. Cells were incubated for 40 h in microaerobic conditions in a defined media as described in Methods. Each assay was performed in triplicate and titres reported if all three samples had observed product production above the limit of quantitation (5 mg l^−1^) (Supplementary Table 2). Error bars show one s.d over the 3 biological replicates.

Consistent with previous reports, the primary products (82% of total alcohol production) with native KIVD are short alcohols (C_2_–C_4_; Fig. 8 and Supplementary Table 2). The KIVD_VLV triple mutant completely switches the product profile of this pathway to predominantly longer-chain (≥C_5_) alcohols at 728 mg l^−1^. Hexanol (C_6_) is the major alcohol detected at a titre of 341 mg l^−1^ (47%), with titres of 269 mg l^−1^ (37%) heptanol (C_7_), and 118 mg l^−1^ pentanol (16%) also observed. GEO 175 also switches the product profile of the pathway so that longer-chain alcohols are the primary products and produced at a level of 522 mg l^−1^. For GEO175, the product profile is further shifted, resulting in heptanol (C_7_) as the major product at a titre of 274 mg l^−1^ (50%), with hexanol production at 160 mg l^−1^ (29%) and 88 mg l^−1^ pentanol (16%). Similar to the results for KIVD_VLV, no ethanol or propanol production is observed, and only 19 mg l^−1^ butanol is produced. In addition, 10 mg l^−1^ of octanol was also observed, a product not observed when either native KIVD or KIVD_VLV were tested under equivalent conditions. The *in vivo* butanol and octanol titres for GEO175 compared with KIVD_VLV are consistent with the *in vitro* enzyme kinetics in which GEO175 has a significantly higher activity on C8, but lower specificity relative to C5, than KIVD_VLV. Total longer-chain alcohol titres for both GEO 175 and KIVD_VLV are increased significantly in comparison with native KIVD, with a >10-fold increase in heptanol production in both cases, and a >95% yield of longer-chain alcohols.

**Figure 9:**
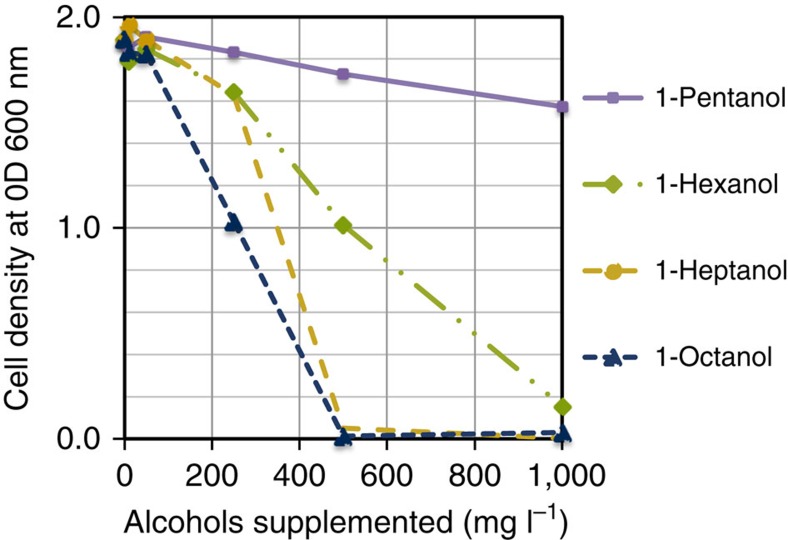
Toxicity as a function of alcohol chain length. The optical density of the engineered *E. coli* strain after a 7-h growth was measured as a function of the alcohol concentration added to growth media at the beginning of incubation.

Replacing KIVD with GEO 175 or KIVD_VLV results in a significant increase in longer-chain alcohol production; however, the overall alcohol production drops ∼10-fold from the 4.0 g l^−1^ obtained with KIVD. We hypothesized that a potential reason for this could be due to the toxicity of longer-chain alcohols. To explore the potential of toxicity being a limiting factor in alcohol production, *E. coli* growth was monitored over a 7-h incubation with 10–1,000 mg l^−1^ C_5_–C_8_ alcohols supplemented at the beginning of growth (Fig. 9). Longer-chain alcohols are observed to be toxic as the supplemented concentration of C_6_–C_8_ alcohols reaches 250 mg l^−1^. The final optical density (OD) of *E. coli* cultures drops from 1.9 to ∼1.6 for C_6_ and C_7_ alcohols, and 1.0 for C_8_. At 500 mg l^−1^ the OD drops to 1.0 and 0.1 for C_6_ and C_7_ alcohols, respectively. No significant growth is observed for C_8_. At 1,000 mg l^−1^ no significant growth is observed for heptanol, and only an OD of 0.2 is achieved in the presence of hexanol. However, growth up to an OD of 1.5 is observed for pentanol. Significant toxicity in the range of 250–1,000 mg l^−1^ for longer-chain alcohols is consistent with the level of longer-chain alcohol titre, roughly 500 mg l^−1^, produced in the engineered *E. coli* strains. This result indicates that to achieve higher total alcohol titre with this pathway, a strain of *E. coli* with high tolerance towards longer-chain alcohols is likely necessary^20^,^21^. However, similar issues with product toxicity have been addressed through the design of continuous extraction methods and could be applied to the system here to increase levels of longer-chain alcohols titres^22^.

## Discussion

In this study we introduce an integrative genomic mining approach to enable the discovery of enzymes for a targeted function from sequence databases. The function of interest here was an enzyme with high activity for the decarboxylation of long-chain ketoacids to modulate the carbon flux of a synthetic pathway and increase titres of longer alcohols. The integrative genomic mining approach led to the discovery of a previously uncharacterized protein that we demonstrate has the targeted functional properties, and only required experimental characterization of 10 new proteins. In parallel, a more traditional computationally directed library-screening approach was utilized to reengineer the activity of a well-established enzyme. This required two successive rounds of screening over 400 mutants against a panel of substrates to identify an enzyme with the desired functional properties. When evaluated for their ability to modulate the carbon flux *in vivo*, both are able to completely shift the product profile towards longer-chain alcohols. However, the genomic mining approach provides a viable alternative to often expensive and laborious enzyme-engineering efforts that require screening libraries of mutants. This new approach for mining protein databases for enzymes of novel function stands to become an increasingly powerful approach as the methods for protein modelling improve and the size of genomic databases grow.

The enzyme GEO 175 represents the product of a general and rapid approach for obtaining enzymes with a desired function from the rapidly growing sequence databases. By combining bioinformatics and molecular modelling, this approach enables the identification of proteins likely to carry out a targeted function regardless of their native or putatively annotated activity. This overcomes issues with misannotation of protein function^23^ or biased assumptions based on the closest characterized protein in the amino-acid sequence. The enzyme identified in this study, GEO175, is a clear illustration of this as it is roughly 15% identical to KIVD and its closest characterized sequence homologue (ppBFD, roughly 50% sequence identity) has a significantly different functional profile.

While GEO175 and KIVD_VLV both increased longer-chain alcohol titre >10-fold, we identified that toxicity needs to be addressed to further increase total longer alcohol titre. The titre of longer-chain alcohols produced is on the order of 0.5 g l^−1^, which we then demonstrate is highly toxic for the cell lines used in this study. For industrial applications, bio-alcohol production often requires titres on the order of over tens of grams per litre (ref. 24). To avoid the need of a continuous extraction fermentation system, future efforts for increasing longer-chain alcohol production should focus on engineering or finding *E. coli* strains that are tolerant to this level of product formation. Efforts to integrate pumps and reengineer the cell wall to be resistant to alcohols could potentially mitigate toxicity effects and concurrently enable higher titres of longer alcohols to be produced through this pathway^20^,^21^.

In addition to toxicity we observed that KIVD_VLV exhibits substrate inhibition kinetics above 2 mM (0.3 g l^−1^) of C8. This could be another potential cause of total alcohol titre loss observed *in vivo*. Redesigning KIVD_VLV to alleviate inhibition would be important for alcohol production at an industrially relevant level with this variant. However, the commensurate specificity and sixfold greater catalytic efficiency possessed by GEO_175 (relative to KIVD_VLV) make it a prime candidate for future industrial strain development for longer-chain alcohol production.

In summary, a new genomic mining approach and computationally directed library design efforts were both successfully implemented to obtain enzymes that enabled specific production of longer-chain alcohols *in vivo*. These results demonstrate the modularity of the synthetic +1 recursive pathway and provided a clear path forward to engineer the industrial-level production of longer alcohols. Finally, the integrative genomic mining approach introduced here is highly general, and with the rapid growth of sequence databases it has the potential to revolutionize the development and discovery of enzyme catalysts.

## Methods

### Integrative genomic mining

To obtain the GEO sequences, the native KIVD sequence was input for a homologous sequence search using HMMER3's* online server^13^. The resulting sequences were filtered using the CD-HIT* online server with a 90% identity cutoff^15^,^25^ (Supplementary Figs 7 and 8). A homology model of each sequence was made using Rosetta Comparative Modeling^14^. At this point, the intermediate was placed into the active site, and 1,000 simulations were run to relax the intermediate according to the constraints. For each model, the lowest 100 in overall protein energy models were selected and then from that subset the lowest protein–ligand interface was chosen as the energy for the GEO.

An intermediate of the C8 product-yielding reaction was modelled using Spartan* (ref. 26). This intermediate is modelled based on the synthetic TPP analogue from a crystal structure of KIVD (PDB ID: 2VBG), which mimics the initial nucleophilic attack by the thiazolium. The six-carbon alkyl chain and the acid were added to the structure of the TPP analogue within Spartan. Different conformations of the alkyl chain were included in the modelling and a conformational library was made using OpenEye Omega* (ref. 27). For the enzyme design of KIVD, this intermediate was placed into the active site using distance and angle constraints. Rosetta Enzyme Design was run with default settings, an example of which is provided in the Rosetta Molecular Modeling Suite demos.

From the design simulations for each GEO, the lowest protein–ligand interface Rosetta energy was used to select a tractable number of GEOs for experimental characterization. During these simulations, any amino acids with a *C*_α_ within 8 Å of the active site could be mutated to any of the 20 amino acids. Ten GEOs of significant interest were chosen with the following criteria: five GEOs were chosen because they had the lowest predicted energies; the second five were chosen with the purpose of maximizing sequence space diversity. The 234 GEOs (less the five lowest in energy) were filtered with a sequence identity cutoff of 40%, and the five sequences from this filtered list with the lowest energy were picked as the second five GEOs in our final list. Each model was evaluated in the Foldit interface, and mutations made during the design simulations were reverted to the native amino acid if not predicted to improve the interface energy by more than two Rosetta energy units.

Phylogenetic tree of GEOs was generated with the Geneious software* using a Muscle sequence alignment^28^*. The resulting tree was visualized using iTOL online tool in the circular tree mode and rooted at native KIVD^29^,^30^.

Synthetic genes coding for each GEO were synthesized as a DNA String by Life Technologies or the Joint Genome Institute. Genes were codon-optimized for *E. coli*, and the DNA as well as amino-acid sequence provided in Supplementary Tables 3 and 4. The string was cloned into the pET-29b(+) plasmid vector using the Gibson assembly between the NdeI and XhoI restriction sites that added a C-terminal 6x-His tag in-frame to the gene.

*Default settings were used unless otherwise specified.

### KIVD active-site redesign

In the design simulations, 10 residues in the proposed active-site pocket were allowed to either remain native or sample any of 11 relatively hydrophobic amino acids (Val, Leu, Ile, Met, Phe, His, Gly, Ala, Thr, Tyr and Trp). The identities of amino acids at all other positions were kept constant. Residues within 12 Å of the ligand were allowed to undergo conformational sampling during simulation. A total of 10,000 design simulations were run, from which the 50 designs lowest in ligand–protein interface energy, and non-redundant in terms of sequence, were selected to represent the potential sequence space predicted to accommodate the C8 substrate. The profile was used to construct a small library of ∼400 KIVD mutants. Each amino acid in the library was sampled as a single mutation, with the exception of residues that were within five residues from one another. These were sampled in a combinatorial manner as both single and double mutants to evaluate synergistic effects, given their proximity to one another. A complete list of oligos and the amino acids allowed for each site are provided in the Supplementary Tables 5 and 6.

### Construction and selection of KIVD libraries

All *E. coli* strains and plasmids used are listed in Supplementary Table 7. Partially degenerate oligonucleotides (Supplementary Table 6) were ordered from Integrated DNA Technologies (San Diego, CA) and were used to generate libraries of *kivd* mutants (with N-terminal histidine tags) using PCR. Library DNA was purified, inserted into the PCR-amplified pQE9 vector by isothermal Gibson assembly^31^ and transformed into XL1-Blue cells. Individual ampicillin-resistant colonies were picked and grown in 96-well blocks at 37 °C overnight. The number of colonies picked was three times the theoretical library size to ensure ∼95% probability of all possible mutation combinations occurring. Glycerol was added to 25% (w/v) and libraries were stored at −80 °C until enzyme activity and specificity were assayed. KIVD mutant libraries were screened using a pH-monitored enzyme assay (method explained below) to measure the rate of H^+^ consumption resulting from the decarboxylation of 2-ketoacids.

KIVD mutant libraries were grown overnight in culture blocks, diluted 1:100 into fresh media (Luria Broth), grown at 37 °C for 3 h to OD_600_ ∼0.6, induced with 0.1 mM isopropyl-b-D-thiogalactoside (IPTG; GoldBio, Saint Louis, MO) and grown for two additional hours at 37 °C. Cells from the well blocks were transferred to 96-well assay plates (Costar, Corning, NY) and cell density was measured at 600 nm. An equal volume of permeabilization solution (8.7 mM potassium phosphate, 43.4 mM KCl, 0.87 mM MgSO_4_, pH 7.1±0.1, 8.7% (v/v) chloroform, 0.0043% (w/v) SDS and 0.26% (v/v) 2-mercaptoethanol) was then added to break the cell membranes. Bromothymol blue, TPP (Sigma-Aldrich, St Louis, MO) and substrate were added to 0.008% (w/v), 0.5 and 10 mM, respectively. All substrates (2-ketobutyrate, 2-ketovalerate, 2-ketoisovalerate, 2-ketocaproate and 2-ketooctanoate; Sigma-Aldrich) were dissolved in MilliQ dH_2_O and pH was adjusted to 7.1±0.1. Absorbance was measured at 615 nm in a spectrophotometer (TEK Powerwave XS, BioTek, Winooski, VT). Data were acquired for 15 min at 30 °C. Cells containing pQE_hiskivd_wt and pQE9 were used as positive and negative controls, respectively. Enzyme activity data were corrected for cell density. Mutants chosen from this screening had a 25% higher activity than wild-type KIVD for any of the 2-ketoacids.

The library derived from the pH-coupled assay was further screened using a second enzyme kinetics assay. This assay involved a second enzyme, ADH from *S. Cerevisiae* (ADH6), which is a key enzyme in the alcohol production pathway in reducing the aldehyde produced by KIVD into an alcohol. ADH couples oxidation of NADPH to NADP^+^ to the reduction of aldehyde into an alcohol. Stoichiometrically, conversion of 1 M of 2-ketoacid to n-alcohol depletes exactly 1 M of NADPH. Therefore, activity of KIVD could be measured directly by measuring depletion of NADPH through ultraviolet spectrophotometry.

Overnight cultures of XL1B with pQE9 containing KIVD mutations were grown at 37 °C in a 96-well block. Cultures were diluted 1:100 and then grown for 3 h at 37 °C to OD_600_ of 0.6. Cultures were induced with 0.1 mM IPTG (GoldBio) for 3 h at 37 °C. Cultures were then centrifuged, and pellets were lysed with BugBuster (Novagen, Madison, WI). Cell lysate (5 μl) from each culture and a buffer mix (175 μl) containing coenzyme 1.5 mM TPP (Sigma), 0.2 mM NADPH (Fisher Scientific, Waltham, MA), 0.045 U ADH6, 100 mM NaPO_4_, 100 mM NaCl and 10 mM MgCl_2_, pH of 7, were added together into a 96-well plate. In all, 20 μl of 100 mM substrate was added to dilute to make a final concentration of 10 mM. The substrates tested were IsoC5, C4, C5, C6, C8 and H_2_O (Supplementary Fig. 2). Absorbances were measured with a plate reader (TEK Powerwave XS, BioTek) at 340 nm at 30 °C for 15 min. The protein concentration in each sample was determined with a BCA assay (Thermo Scientific, Waltham, MA) and used to normalize slope values. Candidate KIVD mutants were chosen for greater activity on long-chained 2-ketoacids and decreased activity on shorter 2-ketoacids. This was determined from graphing absorbance versus time for each substrate and qualitatively comparing slope values versus wild type (Supplementary Fig. 2).

### Site-directed mutagenesis

Oligonucleotides (Supplementary Table 6) encoding specific *kivd* mutations were ordered from Integrated DNA Technologies (Coralville, Iowa) and were used to mutate *kivd* in pZE_LeuA*BCDKA6 and pQE_hiskivd_wt using PCR. Amplified DNA fragments were purified, inserted into either the PCR-amplified pZE vector containing leuA*BCD and *adh6* or the pQE vector by isothermal Gibson assembly, and transformed into XL1-Blue cells. Plasmid DNA was purified (Qiagen, Hilden, Germany) from overnight cultures of antibiotic-resistant colonies and the plasmid sequences were verified (Laragen, Culver City, CA).

### Chromosomal gene knockout

Genes were removed from the ATCC 98082 *ΔrhtA* strain genome using P1 transduction from the Keio collection as previously described^32^. The aldehyde-ADH gene (*adhE*) was knocked out to eliminate ethanol production from acetyl-coA. In all strains, *recA* was knocked out to prevent recombination between the genome and plasmids, thereby stabilizing the transformants. Primers used to target *recA* for knockout (Supplementary Table 6) were designed based on the Keio collection (Genobase, http://ecoli.aist-nara.ac.jp/) and ordered from Integrated DNA Technologies.

### Fermentation procedure and analysis

For *n*-alcohol production, strains of ST128 were transformed with pZS_thrO, pZAlac_ilvA_BS_leuA and pZE_LeuA*BCDK*A6 containing various *kivd* mutations. Fermentation conditions were adapted from those in previous lines of works^3^,^4^, with the following changes: 20 ml of medium was used, with 100 μg ml^−1^ ampicillin, 50 μg μl^−1^ kanamycin and 100 μg ml^−1^ spectinomycin added. Cells were grown to an optical density at 600 nm of ∼0.6, followed by induction with 0.1 mM IPTG. After fermentation, cells were centrifuged for 15 min at 4,000*g* and 4 °C. The supernatant was split into two fractions for analysis, 5 ml for short-chain alcohols (ethanol, 1-propanol and 1-butanol) and 15 ml for longer-chain alcohols (1-pentanol, 1-hexanol, 1-heptanol and 1-octanol). Longer-chain alcohols were extracted from the 15-ml fraction by 3 ml *n*-hexane before analysis. GC-FID analysis was performed as previously described^4^.

### Protein purification and enzymatic assay of KIVD

For the native KIVD and KIVD_VLV mutant, 2 ml overnight cultures of XL1B cells were transformed with pQE9 containing N-terminal his-tagged KIVD enzymes and grown in Terrific Broth (BP Biomedical, Cat# 3046-042) with 50 μg ml^−1^ of carbenicillin (Fisher Scientific, Cat# BP2648-5). For the GEOs, 2-ml overnight cultures of BLR cells were transformed with pet29b+ plasmid containing N-terminal his-tagged GEOs, and grown in Terrific Broth with 50 μg ml^−1^ of kanamycin (Fisher Scientific, Cat# BP906-5). These cultures were diluted 1:1,000 in 500 ml of Terrific Broth with 1 mM MgSO_4_, 1% glucose and 50 μg ml^−1^ of corresponding antibiotics, and then grown at 37 °C for 24 h. Cultures were pelleted down at 4,700 r.p.m. for 10 min and resuspended in auto-induction media (LB broth, 1 mM MgSO_4_, 0.1 mM TPP, 1 × NPS and 1 × 5052) for induction at 18 ^o^C for 34 h. At the end of induction, cells were centrifuged (4,700 r.p.m., 4 °C, 20 min), supernatant was removed and cells were resuspended in 40 ml lysis buffer (100 mM Hepes, pH 7.5, 100 mM NaCl, 10% glycerol, 0.1 mM TPP, 1 mM MgSO_4_, 10 mM Imidazole, 1 mM TCEP) and 1 mM phenylmethylsulphonyl fluoride and sonicated for 2 min. Lysed cells were centrifuged at 4,700 r.p.m. at 4 ^o^C for 60 min to remove cell debris. Supernatant was loaded on gravity flow column with 700 μl of cobalt slurry (Fisher Scientific, CAT# PI-90091) washed with 10 ml of wash buffer (100 mM Hepes, pH 7.5, 100 mM NaCl, 10% glycerol, 0.1 mM TPP, 1 mM MgSO_4_, 10 mM Imidazole and 1 mM TCEP). Cobalt bead bed was washed with 15 ml of wash buffer five times and proteins were eluted with 1,000 μl of elution buffer (100 mM Hepes, pH 7.5, 100 mM NaCl, 10% glycerol, 0.1 mM TPP, 1 mM MgSO_4_, 200 mM Imidazole and 1 mM TCEP). Protein samples were immediately buffer-exchanged with spin concentrators (Satorius, CAT# VS0112) into storage buffer (100 mM Hepes, pH 7.5, 100 mM NaCl, 10% glycerol, 0.1 mM TPP, 1 mM MgSO_4_ and 1 mM TCEP) and stored at 4 ^o^C until kinetics characterization. Protein concentrations were determined using a Synergy H1 spectrophotometer (Biotek) by measuring absorbance at 280 nm using their calculated extinction coefficients with the ExPASy ProtParam Tool^33^. All other buffers and salts were purchased from Fisher Scientific, unless otherwise specified.

The *k*_cat_ and *K*_M_ values of selective KIVD mutants were measured for the substrates C_3_, isoC_5_ and C_8_. All substrates were dissolved in MilliQ H_2_O and pH was adjusted to 7.5 as necessary. Activity was measured at 0.005–10 mM substrates. The assay was performed in a 96-well half-area plate. Each reaction contains a final concentration of 0.5 mM NADH, 1 mM dithiothreitol, 0.1 mM TPP, 1 mM MgSO4, reaction buffer (100 mM Hepes, 100 mM NaCl, 10% glycerol, pH 7.5) and ADH (Sigma-Aldrich, A7011, 10 U ml^−1^ for C_3_, C_5_ and C_8_ reactions, 500 U ml^−1^ for isoC_5_ reactions). A wide range of ketoacid decarboxylase concentrations, 4.5 nM–15 μM, was used according to the activity of each enzyme towards different substrates to perform steady-state kinetics measurement over a period of an hour. Absorbance readings were taken every 1 min at OD_340_ at 21 °C for 60 min using the Synergy H1. Kinetic parameters (*k*_cat_ and *K*_M_) were determined by fitting initial velocity versus substrate concentration data to the Michaelis–Menten equation.

### Alcohol toxicity

Alcohol tolerance of the *in vivo* alcohol production strain was evaluated by supplementing the fermentation media with specified quantities of longer-chain alcohols, and measuring growth over 7 h. Both the *E. coli* strain (ATCC 98082 pZS_thrO, pZAlac_ilvA_LeuA and pZE12LeuA*BCDKA6_KIVD_wt) and fermentation media (1X M9 metals+1X trace metal mix+0.5% yeast extract+2% glucose+antibiotics) and conditions are the same as described above (Fermentation procedure and analysis). Cells were grown in media without IPTG induction to a starting OD_600_∼0.02 and then supplemented with either 1-petanol, 1-hexanol, 1-heptanol or 1-octanol at specified concentrations of 10, 50, 250, 500 or 1,000 mg l^−1^. OD_600_ readings were taken hourly up to 7 h. Figure 9 shows the final OD_600_ recorded at 7 h.

## Additional information

**How to cite this article:** Mak, W. S. *et al.* Integrative genomic mining for enzyme function to enable engineering of a non-natural biosynthetic pathway. *Nat. Commun.* 6:10005 doi: 10.1038/ncomms10005 (2015).

## Supplementary Material

Supplementary Information Supplementary Figures 1-8 and Supplementary Tables 1-7

## Figures and Tables

**Figure 1:**
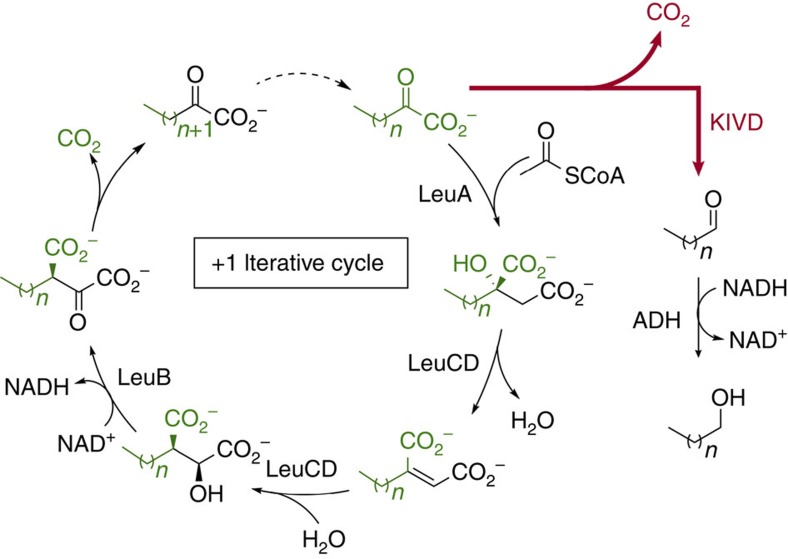
The synthetic recursive +1 pathway. This pathway employs enzymes LeuABCD from leucine biosynthesis for carbon-chain elongation of 2-ketoacids. The enzyme KIVD performs the decarboxylation of 2-ketoacids and diverts carbon out from the +1 iterative cycle.

**Table 1:** Enzyme kinetic constants of native, designed and discovered ketoacid decarboxylases.

***k***_**cat**_**/*K***_**M**_**(M**^−1^** s**^−1^**)**	**C3**	**C5**	**C8**	**iso C5**	
GEO179	0.9±0.02	100±15	1,200±130	41±1.2	Discovered
GEO195	2.4±0.1	200±14	1,400±160	8±0.3	Discovered
GEO175	0.51±0.02	48±3.7	17,000±2,700	3.3±0.2	Discovered
GEO175 L376T_T240S	0.27±0.02	30±7.2	1,100±160	1.6±0.1	Designed
1OVM	32±0.5	2,100±420	80,000±4,300	1,300±130	Native
2VBI	5,700±1,400	52±1.8	14±0.8	1.8±0.1	Native
3FZN	5.4±0.1	1,700±110	350±30	110±10	Native
1ZPD	8,200±545	140±5.6	19±2	0.33±0.01	Native
1OZF	n.d.	0.53±0.04	n.d.	17±0.9	Native
Native KIVD	42±0.6	9,500±470	32,000±5,500	14,000±1,100	Native
KIVD_VLV	0.71±0.07	1.3±0.2	2,800±860	0.24±0.02	Designed
					
*k*_*cat*_ (s^−1^)					
GEO179	n.d.	0.47±0.03	0.32±0.01	0.39±0.01	
GEO195	n.d.	0.49±0.01	0.56±0.02	0.072±0.001	
GEO175	n.d.	0.97±0.04	10.1±0.6	n.d.	
GEO175 L376T_T240S	n.d.	0.28±0.04	3.4±0.2	n.d.	
1OVM	0.2±0.01	1±0.1	1.7±0.1	7.8±0.4	
2VBI	25±2.6	n.d.	0.051±0.01	0.025±0.001	
3FZN	n.d.	4.7±0.1	1.3±0.1	0.52±0.02	
1ZPD	46.7±1.4	0.95±0.02	0.021±0.001	n.d.	
1OZF	n.d.	n.d.	n.d.	0.03±0.001	
Native KIVD	n.d.	14.3±0.2	7±0.3	61±2.1	
KIVD_VLV	n.d.	0.013±0.001	0.5±0.03	n.d.	
					
*K*_M_ (mM)					
GEO179	n.d.	4.6±0.6	0.27±0.03	9.4±0.2	
GEO195	n.d.	2.5±0.2	0.4±0.04	9.1±0.3	
GEO175	n.d.	20±1.3	0.58±0.09	n.d.	
GEO175 L376T_T240S	n.d.	10±2	3.1±0.4	n.d.	
1OVM	7.5±0.1	0.5±0.1	0.021±0.001	6.1±0.6	
2VBI	4.3±1.0	n.d.	3.6±0.2	14±0.5	
3FZN	n.d.	2.7±0.2	3.8±0.3	4.9±0.3	
1ZPD	5.7±0.3	7.1±0.3	1.2±0.1	n.d.	
1OZF	n.d.	n.d.	n.d.	1.8±0.1	
Native KIVD	n.d.	1.5±0.1	0.21±0.04	4.5±0.3	
KIVD_VLV	n.d.	10±1	0.18±0.05	n.d.	

The kinetic constants were determined, as described in Methods, against 2-ketooctanoate (C8), 2-ketoisovalerate (isoC5) and pyruvate (C3). The curves consisted of at least five points. The limit of detection under the conditions tested was 0.2 M^−1^ s^−1^, kinetic constants beyond our detection limit are labeled as n.d. (not determined). Michaelis–Menten curve fits are shown in Supplementary Figs 3–6. Errors are shown as ±one s.d.
