# Corrigendum: Integrative genomic mining for enzyme function to enable engineering of a non-natural biosynthetic pathway

**DOI:** 10.1038/ncomms11912

**Published:** 2016-06-07

**Authors:** Wai Shun Mak, Stephen Tran, Ryan Marcheschi, Steve Bertolani, James Thompson, David Baker, James C. Liao, Justin B. Siegel

Nature Communications 6: Article number: 10005 10.1038/ncomms10005 (2015); Published: 11242015; Updated: 06072016

In the original version of this Article the chemical structures of 2-ketoisovaleric acid and isobutanal in Fig. 3a were incorrect. The correct version of Fig. 3 appears below.


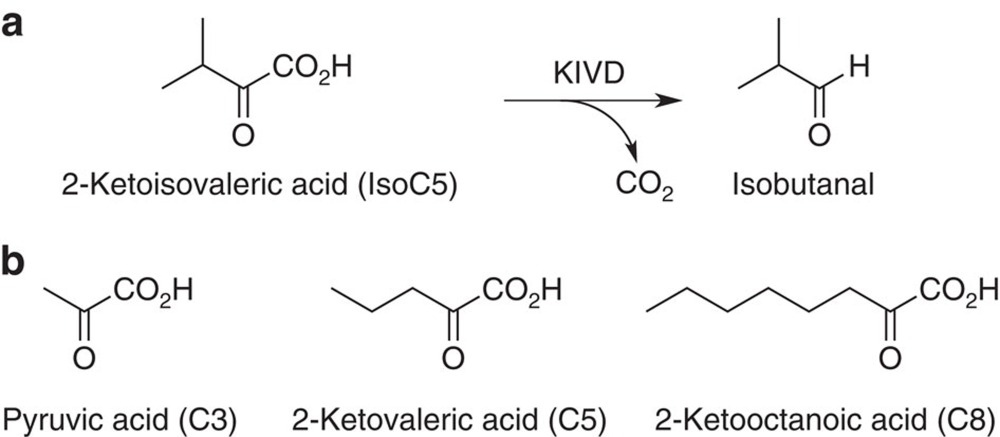


**Figure 3**

