# Supplementary material for: Integrative genomic mining for enzyme function to enable engineering of a non-natural biosynthetic pathway
Source: Nat Commun. 2015 Nov 24;6:10005. doi: 10.1038/ncomms10005 (PMC4673503; doi:10.1038/ncomms10005)
Supplement: Supplementary Information — Supplementary Figures 1-8 and Supplementary Tables 1-7 [file ncomms10005-s1.pdf]

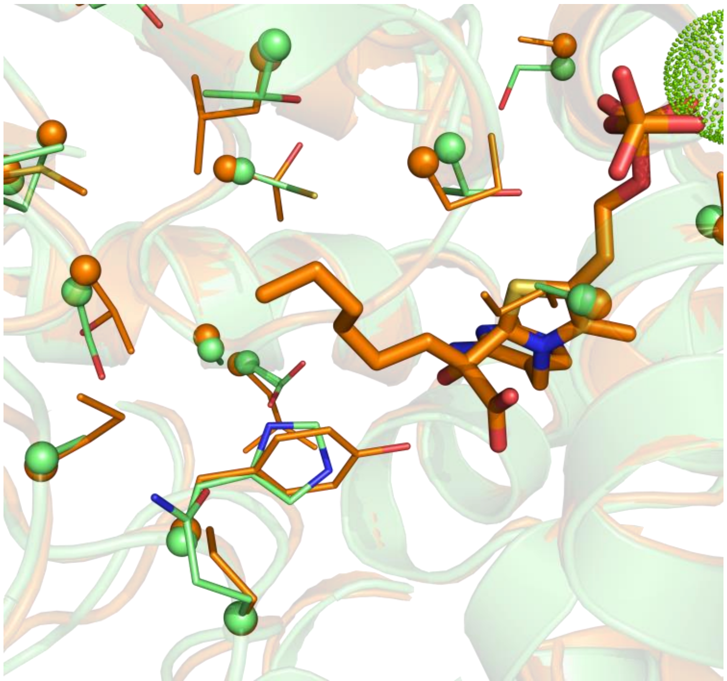

Supplementary Figure 1. Overlay of active sites between GEO 175 (orange) and BFD (green). Residues that are different between the proteins are shown in sticks and their corresponding alpha carbons are shown as spheres.

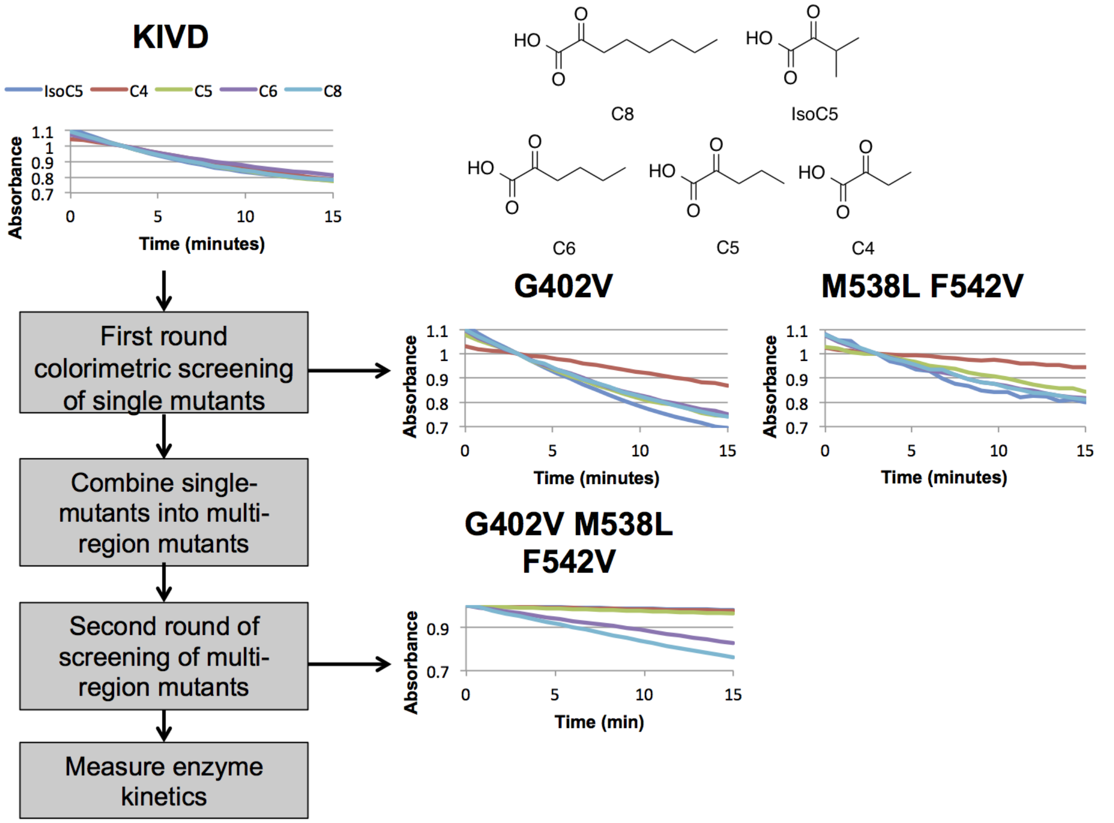

Supplementary Figure 2. Screening process for identifying engineered KIVD with altered substrate specificity. Pertinent screening results/candidate mutants are shown (graphs) which contributed directly to finding KIVD_VLV. Colorimetric assays (graphs) measured approximate enzymatic activity of screened mutants for ketoisovalerate (IsoC5), 2-ketobutyrate (C4), 2-ketovalerate (C5), 2-ketohexanoate (C6), and 2-ketooctanoate (C8). Higher activity is shown by larger downwards slope of [Absorbance]/[Minute]; greater specificity, relative Kivd, was observed in mutants with reduced activity (smaller downward slope) for IsoC5, C4, and C5, and only slightly affected or greater activity (similar or larger downward slope) for C6 and C8. First round high-throughput colorimetric screening of single mutants identified candidates such as G402V and M538L/F542V. Mutations from each region that showed potential in altering the specificity of KIVD were then combined to produce multi-region mutants through PCR-site directed mutagenesis using the plasmids of KIVD single region mutants as templates. These KIVD multi-region mutants, such as G402V M538L F542V (KIVD_VLV) were then screened a second time with the colorimetric assays. Enzyme kinetics was then measured for mutant KIVD_VLV.

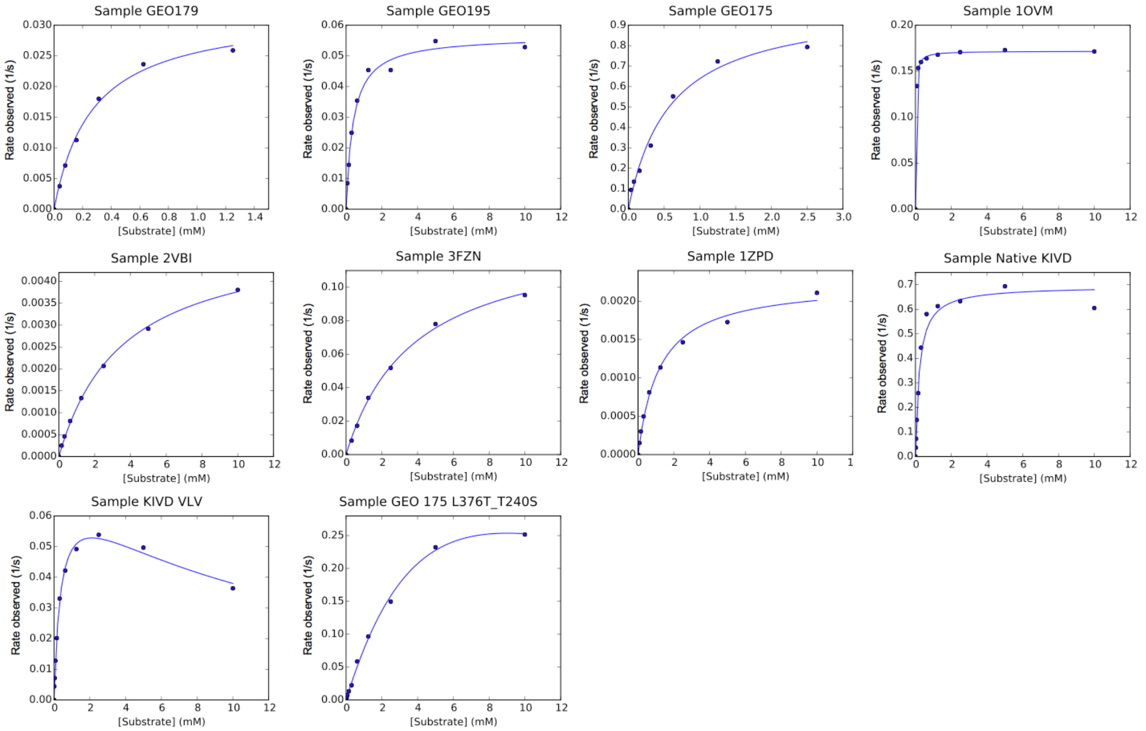

Supplementary Figure 3. Michaelis-Menton curve fit of tested enzymes with C8 substrate. The mutant KIVD_VLV shows inhibition kinetics under high concentration of C8.

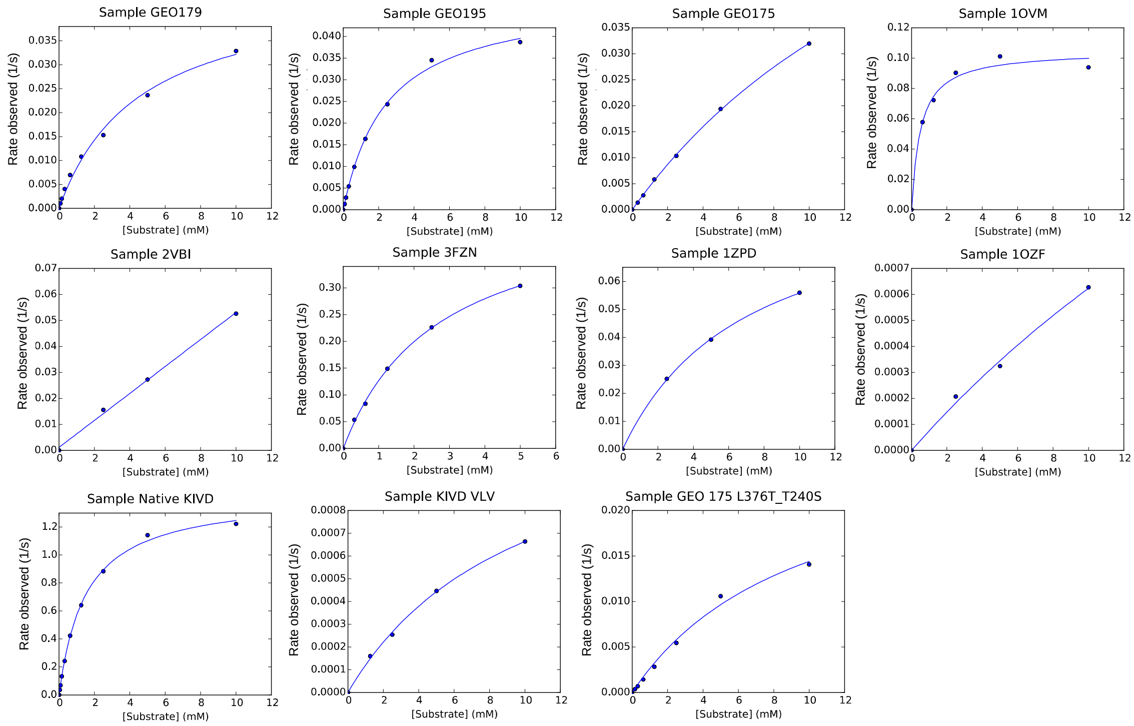

Supplementary Figure 4. Michaelis-Menten curve fits of tested enzymes with C5 substrate.

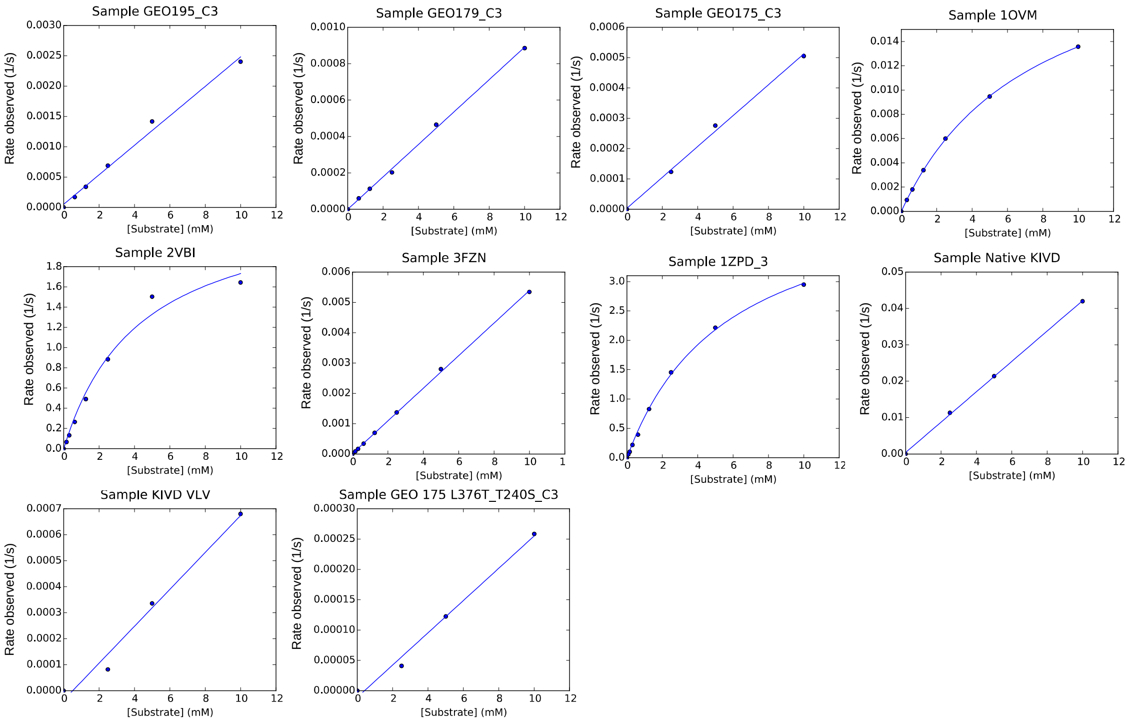

Supplementary Figure 5. Michaelis-Menten curve fits of tested enzymes with C3 substrate.

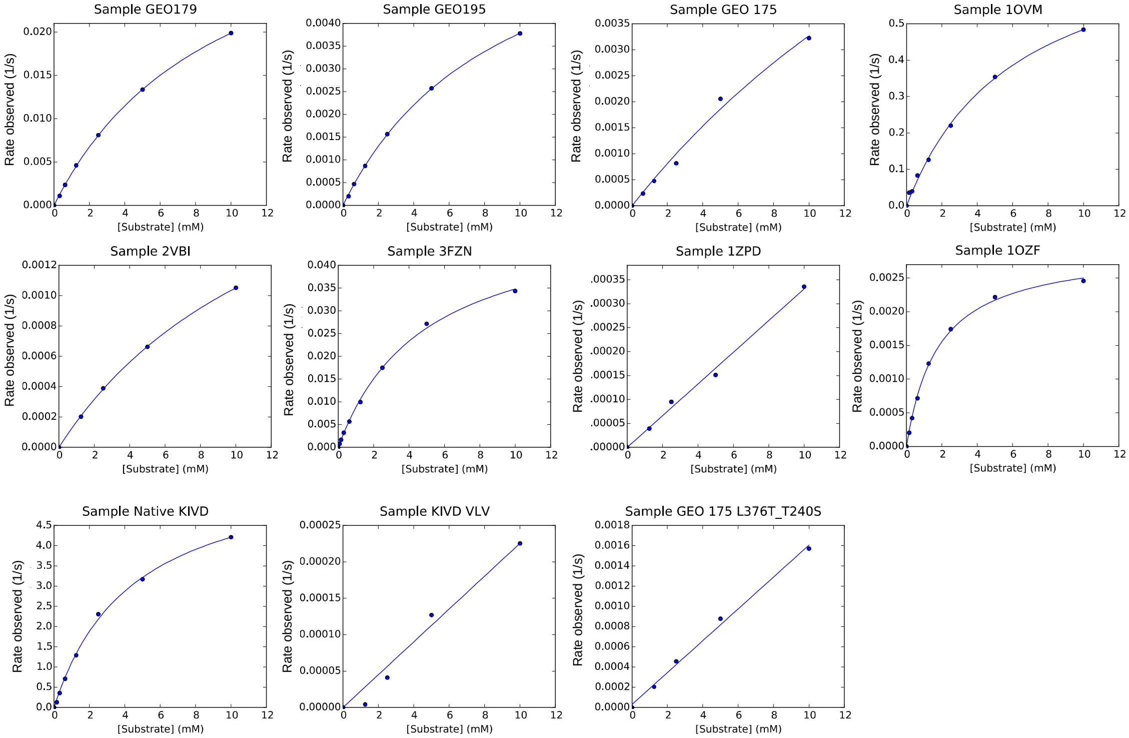

Supplementary Figure 6. Michaelis-Menten curve fits of tested enzymes with isoC5 substrate.

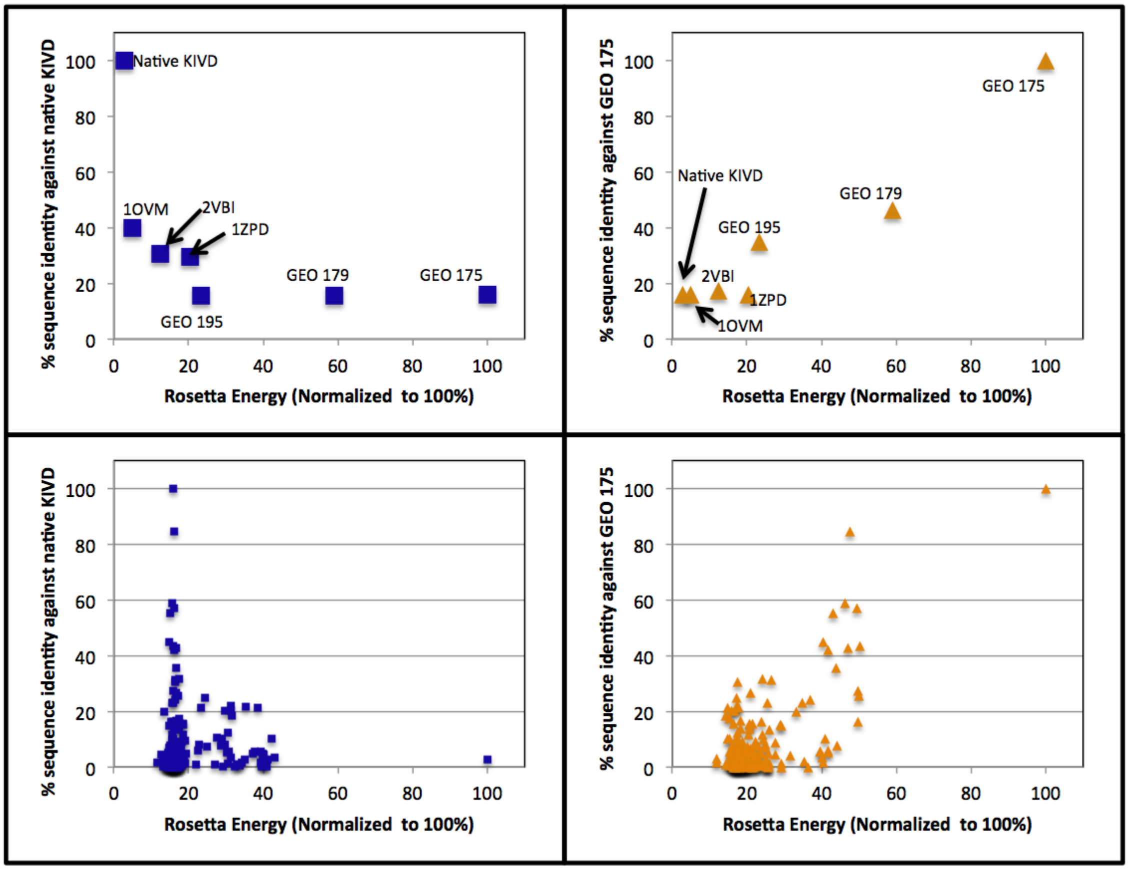

Supplementary Figure 7. % sequence identity of all 239 KIVDs (against native KIVD on the left; against GEO 175 on the right) plotted against the interface energy, calculated from Rosetta docking simulations.

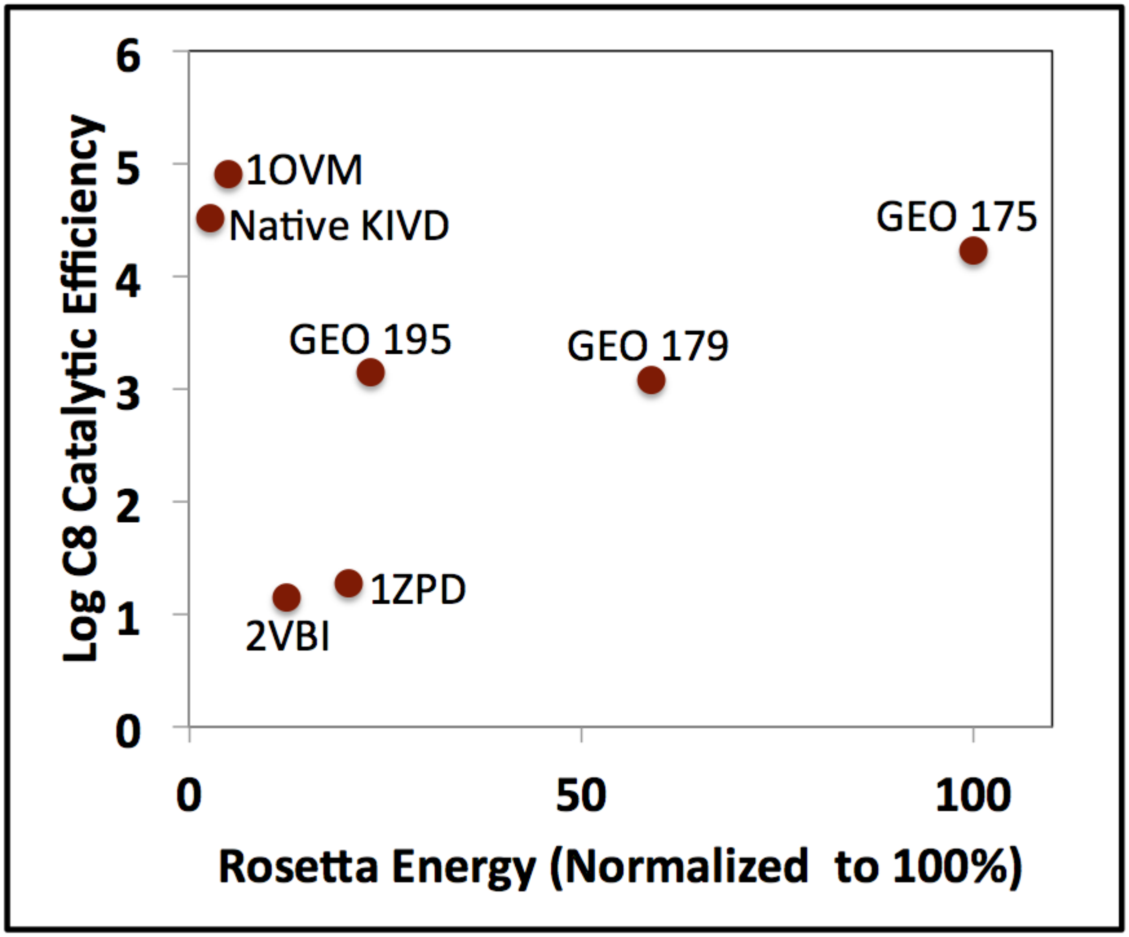

Supplementary Figure 8. Log C8 catalytic efficiency plotted against the interface energy calculated from Rosetta docking simulations.

**1**	2VBG	**41**	ZP_08570611	**81**	YP_001208174	**121**	ADP12107	**161**	O53554	**201**	YP_001070609
**2**	YP_004211875	**42**	YP_001236700	**82**	YP_830466	**122**	YP_004115182	**162**	YP_003909136	**202**	YP_004418205
**3**	YP_004506975	**43**	YP_001208817	**83**	YP_675840	**123**	ZP_08465980	**163**	ZP_08198305	**203**	YP_675476
**4**	ZP_07949728	**44**	YP_002353797	**84**	ZP_06842871	**124**	ZP_06711201	**164**	ZP_07042487	**204**	YP_675461
**5**	ZP_07380174	**45**	2Q5Q	**85**	ZP_05126709	**125**	ZP_08142152	**165**	YP_004210411	**205**	ZP_05074445
**6**	YP_004116674	**46**	YP_002379932	**86**	ZP_05094980	**126**	YP_511062	**166**	YP_925359	**206**	YP_004524050
**7**	ZP_08307744	**47**	O33112	**87**	ZP_07031890	**127**	ZP_07657182	**167**	CAE38656	**207**	ZP_06822743
**8**	ZP_08461032	**48**	P0A623	**88**	YP_004416763	**128**	ZP_07356713	**168**	ZP_01901192	**208**	ZP_01044868
**9**	1OVM	**49**	ZP_04998899	**89**	EGD05600	**129**	ZP_05100555	**169**	ZP_07973795	**209**	ZP_05844198
**10**	ADP13207	**50**	YP_004291227	**90**	ZP_08543603	**130**	YP_001240610	**170**	CAM60234	**210**	ZP_07985939
**11**	YP_001281252	**51**	YP_001353840	**91**	ZP_06271645	**131**	YP_001237056	**171**	ZP_06711620	**211**	BAJ49000
**12**	YP_001177638	**52**	3D7K	**92**	ZP_06273171	**132**	AAM12352	**172**	YP_004020384	**212**	ZP_01626788
**13**	O53865	**53**	YP_004519441	**93**	ABI47993	**133**	YP_001768986	**173**	YP_002133221	**213**	ZP_06710664
**14**	ZP_04562872	**54**	CAP40738	**94**	ZP_07282849	**134**	YP_004216045	**174**	YP_001378281	**214**	YP_509362
**15**	AAL18557	**55**	ZP_08275845	**95**	YP_001208015	**135**	YP_001280485	**175**	ZP_07290467	**215**	YP_004076523
**16**	Q9CBD6	**56**	YP_004523976	**96**	ZP_06370153	**136**	ZP_00956146	**176**	BAD32743	**216**	ZP_08631155
**17**	YP_003731250	**57**	ZP_07706802	**97**	ZP_05125236	**137**	ZP_05033599	**177**	YP_004522883	**217**	AAR37826
**18**	AEG26729	**58**	YP_004081235	**98**	ZP_06757044	**138**	ZP_08591801	**178**	YP_004083736	**218**	ZP_01302664
**19**	ZP_01623010	**59**	ZP_08124815	**99**	YP_830089	**139**	ZP_06412949	**179**	ZP_06547677	**219**	ZP_05001859
**20**	YP_003137934	**60**	ZP_07949857	**100**	YP_004230473	**140**	CAM62618	**180**	ZP_04607738	**220**	CAP43929
**21**	ZP_06368393	**61**	ZP_08303118	**101**	YP_002536327	**141**	CAE37944	**181**	ZP_05135002	**221**	ZP_03267724
**22**	YP_002380104	**62**	YP_987677	**102**	ZP_01130961	**142**	CAP42719	**182**	ZP_08119462	**222**	ZP_06418208
**23**	YP_004674227	**63**	YP_686086	**103**	YP_002377567	**143**	ZP_01999289	**183**	ZP_07280171	**223**	ZP_07216338
**24**	YP_004646634	**64**	ZP_05068817	**104**	YP_001242472	**144**	ZP_08124133	**184**	ZP_07380799	**224**	ZP_01886096
**25**	ZP_08317680	**65**	ZP_06549025	**105**	YP_004075455	**145**	ZP_01902746	**185**	YP_641501	**225**	YP_004695810
**26**	AAM49566	**66**	YP_003810578	**106**	YP_004505036	**146**	YP_003607818	**186**	YP_004085144	**226**	ZP_04997132
**27**	YP_004017390	**67**	YP_004524326	**107**	ZP_07378926	**147**	ZP_07280108	**187**	YP_830320	**227**	ADI17252
**28**	YP_001224592	**68**	ZP_07282256	**108**	ZP_07284792	**148**	ZP_08317504	**188**	ZP_04996569	**228**	ZP_05780727
**29**	2VBI	**69**	BAF52673	**109**	ZP_07278109	**149**	YP_001769586	**189**	CAQ52617	**229**	CAE50484
**30**	ZP_01125207	**70**	YP_003909474	**110**	CAK95977	**150**	ZP_08272344	**190**	YP_001684628	**230**	CAC11911
**31**	YP_004210504	**71**	YP_004212185	**111**	YP_004490314	**151**	ZP_05102608	**191**	AAA17301	**231**	ZP_06273867
**32**	1ZPD	**72**	YP_001279645	**112**	CAK95978	**152**	ZP_01306645	**192**	Q9HUR2	**232**	YP_001275313
**33**	YP_001806325	**73**	CAE45665	**113**	BAC79260	**153**	YP_001505772	**193**	YP_004080965	**233**	YP_001240047
**34**	AAA27685	**74**	YP_831380	**114**	ZP_08387358	**154**	YP_001203581	**194**	AEG30045	**234**	ZP_06712511
**35**	YP_730696	**75**	YP_370152	**115**	ZP_07278993	**155**	ZP_07705403	**195**	ZP_06846103	**235**	ZP_07033476
**36**	YP_003797329	**76**	ZP_05101111	**116**	YP_004230546	**156**	YP_004416777	**196**	ZP_07281735	**236**	YP_004081191
**37**	ZP_08315793	**77**	AAR05436	**117**	ZP_06841705	**157**	ZP_07282273	**197**	YP_047867	**237**	ZP_08333578
**38**	ZP_01079084	**78**	YP_001203568	**118**	ACR33042	**158**	ZP_07674873	**198**	YP_004317430	**238**	ZP_01725425
**39**	ZP_08074854	**79**	YP_381143	**119**	ZP_01749177	**159**	YP_481591	**199**	YP_004534047	**239**	ZP_05285963
**40**	CBE69591	**80**	CAC11945	**120**	ZP_08137686	**160**	NP_484471	**200**	YP_004077246		

Supplementary Table 1. PDB code and accession numbers of GEOs modeled, as listed on the phylogenetic tree depicted in Figure 2. Sequences were obtained from homologous sequence search in the Non-Redundant database using HMMER3. The 10 GEOs chosen for experimental characterization are highlighted (Orange: solubly expressed, blue: did not express).

	Native KIVD (mg/L)	KIVD V_LV (mg/L)	GEO 175 (mg/L)
1-Ethanol	652 ± 32	n.d.	n.d.
1-Propanol	693 ± 4	n.d.	n.d.
1-Butanol	1964 ± 24	n.d.	19 ± 2
1-Pentanol	594 ± 20	118 ± 29	88 ± 12
1-Hexanol	75 ± 6	341 ± 63	160 ± 18
1-Heptanol	20 ± 3	269 ± 23	274 ± 19
1-Octanol	n.d.	n.d.	10 ± 1
Total Alcohol Titer	3998 ± 89	728 ± 115	551 ± 52

Supplementary Table 2. *In vivo* alcohol production. Cells were incubated for forty hours in microaerobic conditions in a defined media as described in Materials and Methods. The best enzyme in shifting the product profile is GEO 175, with 1-heptanol being the major product. Error measurements (mg/L) show one standard deviation over 3 biological replicates.

Supplementary Table 3. DNA sequences for all genes used in this study.

DNA Sequences
3FZN
ATGGCGAGCGTGCATGGCACCACGTATGAACTGCTGCGTCGCCAGGGTATCGATACCGTGTTCGGCAACCCG<br>GGTTCAAATGAACTGCCGTTTCTGAAAGATTTCCCGGAAGACTTTCGTTATATCCTGGCACTGCAAGAAGCG<br>TGCGTGGTTGGCATTGCAGACGGTTACGCGCAAGCCTCGCGCAAACCGGCGTTTATTAACCTGCATAGCGCG<br>GCCGGCACCGGTAATGCAATGGGCGCTCTGAGCAACGCGTGGAACAGCCACAGCCCGCTGATCGTGACCGCG<br>GGCCAGCAAACGCGTGCCATGATTGGTGTGGAAGCACTGCTGACGAACGTTGATGCAGCTAATCTGCCGCGC<br>CCGCTGGTCAAATGGTCCTATGAACCGGCATCAGCGGCCGAAGTGCCGCATGCAATGTCTCGTGCCATCCAC<br>ATGGCAAGTATGGCCCCGCAGGGTCCGGTCTATCTGTCTGTGCCGTACGATGACTGGGATAAAGACGCCGAT<br>CCGCAGAGTCATCACCTGTTTGATCGTCATGTTAGCTCTAGTGTCCGCCTGAACGACCAGGATCTGGATATC<br>CTGGTTAAAGCACTGAACTCTGCTAGTAATCCGGCGATTGTGCTGGGTCCGGATGTTGACGCAGCTAACGCA<br>AATGCTGATTGCGTGATGCTGGCTGAACGTCTGAAAGCGCCGGTTTGGGTCGCACCGTCGGCTCCGCGTTGC<br>CCGTTCCCGACCCGTCACCCGTGTTTTCGTGGTCTGATGCCGGCCGGTATTGCAGCAATCAGCCAGCTGCTGG<br>AAGGCCATGATGTCGTGCTGGTCATCGGTGCACCGGTGTTCCGCTATCACCAGTACGACCCGGGCCAATATC<br>TGAAACCGGGTACCCGTCTGATTTCTGTTACGTGTGATCCGCTGGAAGCAGCTCGCGCGCCGATGGGCGATG<br>CAATCGTGGCAGACATTGGTGCGATGGCCAGTGCACTGGCTAACCTGGTTGAAGAATCCTCACGTCAGCTGC<br>CGACCGCGGCCCCGGAACCGGCTAAAGTTGATCAAGACGCAGGTCGTCTGCACCCGGAAACCGTCTTTGATA<br>CGCTGAATGACATGGCCCCGGAAAACGCAATTTACCTGAATGAATCCACGTCAACCACGGCCCAGATGTGGC<br>AACGTCTGAACATGCGCAATCCGGGTTCTTATTACTTCTGTGCAGCTGGCGGTCTGGGTTTTGCACTGCCGG<br>CGGCAATCGGTGTGCAGCTGGCGGAACCGGAACGTCAAGTGATTGCCGTTATCGGCGATGGTAGCGCCAACT<br>ATTCGATTAGCGCACTGTGGACCGCAGCTCAGTACAATATTCCGACGATCTTCGTTATTATGAACAATGGCA<br>CCTATGGTGCCCTGCGTTGGTTTGCAGGTGTGCTGGAAGCTGAAAACGTTCCGGGCCTGGATGTCCCGGGTA<br>TCGACTTCCGTGCACTGGCAAAAGGCTACGGTGTTCAGGCACTGAAAGCTGATAATCTGGAACAGCTGAAAG<br>GCTCGCTGCAAGAAGCGCTGAGCGCCAAAGGTCCGGTGCTGATTGAAGTCTCTACCGTGAGTCCGGTTAAAG<br>GCTCCACGGAAAATCTGTATTTTCAGTCAGGTGCGCTCGAG
2VBI
ATGACCTATACGGTGGGCATGTACCTGGCTGAACGCCTGGTGCAGATTGGCCTGAAACATCACTTTGCGGTG<br>GCTGGCGATTACAACCTGGTGCTGCTGGATCAACTGCTGCTGAACAAAGACATGAAACAGATTTATTGCTGT<br>AACGAACTGAATTGCGGCTTTAGCGCAGAAGGTTACGCTCGCTCTAATGGTGCGGCGGCGGCAGTGGTTACC<br>TTCAGTGTGGGTGCCATTTCCGCAATGAACGCTCTGGGCGGTGCTTACGCGGAAAATCTGCCGGTTATTCTG<br>ATCTCAGGCGCGCCGAACTCGAATGATCAGGGCACGGGTCATATCCTGCATCACACCATTGGTAAAACGGAT<br>TATAGCTACCAACTGGAAATGGCACGTCAGGTCACCTGTGCGGCCGAATCAATCACGGATGCGCATTCGGCC<br>CCGGCAAAAATCGACCACGTTATTCGTACCGCACTGCGTGAACGTAAACCGGCATATCTGGATATCGCGTGC<br>AACATTGCAAGCGAACCGTGTGTGCGTCCGGGTCCGGTTAGCTCTCTGCTGAGTGAACCGGAAATTGATCAT<br>ACCTCCCTGAAAGCAGCTGTGGACGCGACGGTTGCCCTGCTGGAAAAATCAGCCTCGCCGGTGATGCTGCTG<br>GGCTCAAAACTGCGTGCAGCAAACGCACTGGCAGCTACCGAAACGCTGGCAGATAAACTGCAGTGCGCTGTG<br>ACCATCATGGCGGCGGCAAAAGGCTTTTTCCCGGAAGATCACGCCGGCTTCCGTGGTCTGTATTGGGGCGAA<br>GTTTCAAATCCGGGTGTCCAGGAACTGGTGGAAACCTCGGATGCACTGCTGTGTATCGCTCCGGTTTTTAAC<br>GACTACAGCACGGTCGGCTGGTCTGCGTGGCCGAAAGGTCCGAATGTGATTCTGGCCGAACCGGACCGTGTT<br>ACCGTCGATGGTCGTGCGTATGATGGTTTTACGCTGCGTGCTTTCCTGCAAGCTCTGGCAGAAAAAGCACCG<br>GCACGTCCGGCTAGTGCACAGAAAAGTTCCGTTCCGACCTGCAGTCTGACCGCGACGTCCGATGAAGCCGGC<br>CTGACGAACGACGAAATCGTTCGCCACATTAACGCGCTGCTGACCAGCAATACCACGCTGGTCGCGGAAACG<br>GGCGATTCTTGGTTCAATGCCATGCGTATGACCCTGCCGCGTGGTGCACGCGTCGAACTGGAAATGCAGTGG<br>GGCCATATTGGTTGGAGCGTGCCGTCTGCATTTGGCAATGCTATGGGTAGTCAGGATCGTCAACACGTCGTG<br>ATGGTGGGCGACGGTTCCTTCCAGCTGACCGCGCAAGAAGTTGCCCAGATGGTCCGTTATGAACTGCCGGTG<br>ATTATCTTTCTGATCAACAATCGCGGCTACGTTATTGAAATCGCCATTCATGATGGTCCGTACAACTACATC<br>AAAAACTGGGACTATGCCGGTCTGATGGAAGTTTTTAACGCAGGCGAAGGTCACGGCCTGGGTCTGAAAGC<br>GACCACGCCGAAAGAACTGACCGAAGCCATTGCACGTGCTAAAGCGAATACCCGCGGCCCGACGCTGATCGA<br>ATGCCAAATTGATCGTACCGACTGTACGGATATGCTGGTCCAGTGGGGTCGCAAAGTGGCGTCTACCAACGC<br>ACGCAAAACGACGCTGGCGGGCTCTACCGAAAATCTGTACTTCCAATCTGGCGCACTCGAG
1ZPD

ATGAGCTATACCGTGGGCACGTACCTGGCTGAACGTCTGGTTCAAATTGGCCTGAAACATCACTTTGCCGTG<br>GCCGGTGATTATAATCTGGTTCTGCTGGACAACCTGCTGCTGAATAAAAACATGGAACAGGTGTACTGCTGT<br>AATGAACTGAACTGCGGCTTCAGTGCGGAAGGTTATGCTCGCGCGAAGGGTGCGGCGGCGGCGGTGGTTACC<br>TACAGTGTTGGTGCCCTGTCCGCATTTGATGCTATCGGCGGTGCCTATGCAGAAAATCTGCCGGTTATTCTG<br>ATCTCCGGCGCCCCGAACAATAACGATCATGCGGCGGGTCATGTCCTGCATCACGCACTGGGTAAAACCGAC<br>TATCATTACCAGCTGGAAATGGCAAAAAACATTACCGCAGCTGCGGAAGCGATCTATACGCCGGAAGAAGC<br>TCCGGCGAAAATTGATCACGTTATCAAAACCGCGCTGCGTGAGAAAAAACCGGTCTACCTGGAAATTGCGTG<br>CAATATCGCCTCAATGCCGTGTGCAGCACCGGGTCCGGCATCGGCACTGTTTAATGATGAAGCAAGCGACGA<br>AGCTTCTCTGAACGCTGCGGTGGATGAAACCCTGAAATTCATTGCGAACCGTGACAAAGTTGCAGTCCTGGT<br>GGGCAGCAAACTGCGTGCCGCAGGTGCAGAAGAAGCTGCGGTCAAATTTACCGATGCACTGGGCGGTGCTGT<br>GGCAACGATGGCCGCAGCTAAAAGCTTTTTCCCGGAAGAAAATGCCCTGTATATCGGCACCTCATGGGGTGA<br>AGTGTCGTACCCGGGTGTTGAAAAAACGATGAAAGAAGCCGATGCAGTCATTGCTCTGGCGCCGGTGTTCAA<br>TGACTATAGCACCACGGGCTGGACCGATATCCCGGACCCGAAAAAACTGGTTCTGGCGGAACCGCGTAGCGT<br>CGTGGTTAACGGTATTCGCTTTCCGTCTGTGCATCTGAAAGATTACCTGACCCGTCTGGCCCAAAAAGTTAG<br>CAAGAAAACCGGCTCTCTGGACTTTTTCAAAAGTCTGAATGCGGGTGAACTGAAAAAAGCAGCACCGGCCG<br>ATCCGTCCGCACCGCTGGTCAATGCGGAAATTGCACGTCAGGTGGAAGCACTGCTGACCCCGAACACCACGG<br>TGATCGCCGAAACGGGCGACTCTTGGTTCAATGCACAACGTATGAAACTGCCGAACGGTGCGCGCGTTGAAT<br>ATGAAATGCAGTGGGGCCATATTGGTTGGAGCGTTCCGGCAGCTTTTGGCTACGCAGTCGGTGCTCCGGAAC<br>GTCGCAACATCCTGATGGTGGGCGATGGTTCGTTCCAGCTGACCGCACAAGAAGTTGCTCAGATGGTCCGTC<br>TGAAACTGCCGGTCATCATCTTTCTGATCAACAACTACGGCTACACGATTGAAGTGATGATCCACGATGGTC<br>CGTATAATAACATCAAAAATTGGGACTACGCCGGCCTGATGGAAGTGTTTAATGGTAACGGCGGTTATGAT<br>AGTGGCGCGGCCAAAGGTCTGAAAGCGAAAACCGGCGGTGAACTGGCCGAAGCAATTAAAGTTGCTCTGGC<br>GAACACCGATGGCCCGACGCTGATTGAATGCTTCATCGGTCGCGAAGACTGTACCGAAGAACTGGTTAAATG<br>GGGCAAACGTGTCGCAGCTGCGAATAGCCGCAAACCGGTGAACAAAGTCGTGGGCAGTACGGAAAACCTGT<br>ATTTTCAGTCCGGTGCGCTCGAG
ZP_08570611
ATGTCATCAATCAACTCGTTCACCGTCGCCGACTACCTGCTGACCCGTCTGCATCAACTGGGCCTGCGTAAG<br>GTTTTTCAAGTGCCGGGCGATTATGTCGCTAACTTTATGGACGCGCTGGAACAGTTCAATGGCATTGAAGCC<br>GTGGGTGATCTGACCGAACTGGGTGCAGGTTATGCGGCCGACGGTTACGCACGTCTGACCGGTATCGGTGCA<br>GTGTCTGTTCAGTTTGGCGTGGGTACGTTTTCTGTTCTGAACGCAATTGCTGGCAGTTACGTTGAACGTAAT<br>CCGGTGGTTGTCATCACCGCGTCGCCGAGCACGGGTAACCGCAAAACCATTAAGGAAACGGGCGTGCTGTTT<br>CATCACTCCACCGGTGATCTGCTGGCTGACTCAAAAGTGTTCGCGAATGTCACGGTGGCAGCTGAAGTTCTG<br>TCTGATCCGAGTGACGCGCGCCAGAAAATTGATAAGGCCCTGACCCTGGCAATTACGTTTCGTCGCCCGATC<br>TATCTGGAAGCCTGGCAGGATGTTTGGGGCCTGGCATGCGAAAAACCGGAAGGTGAACTGAAGGCCCTGCCG<br>CTGATCAGCGAAGAAGGCGCGCTGAAAGCCATGCTGGCAGATTCTCTGAAGCTGCTGAACAGTGCACGTCAG<br>CCGCTGGTTCTGCTGGGTGTCGAAATTAATCGCTTCGGTCTGCAAGATGCTGTTCTGGACCTGCTGAAAGCG<br>TCTGGTCTGCCGTATTCCACCACGTCACTGGCCAAGACCGTTATTAGTGAAAACGAAGGCATCTTTGTCGGC<br>ACCTATGCGGATGGTGCGTCCTTCCCGGCAACGGTGGAATACATCGAAAAAGCCGATTGTGTCCTGGCACTG<br>GGTGTGATTTTTACCGATGACTACCTGACGATGCTGTCAAAACAGTTCGATCAAATGATCGTGGTTAACAA<br>TGACGAAACCTCGCGTCTGGGCCATGCTTATTACCACCAGCTGTATCTGGCGGATTTTATTCTGCAACTGAC<br>GGACGAAATTAAAAAATCTAGCCTGTACCCGCGTCAGAACAGCGCACTGCCGCTGCTGCCGCCGCAACCGCA<br>GATTACCCCGGCGCTGCTGCAACAACAGCTGAGTTATCAGAACTTTTTCGACCTGTTTTATGGTTACCTGCT<br>GCAACATCAGCTGCAAGACAATATTTCCCTGATCCTGGGCGAAAGTTCCTCACTGTATATGTCAGCTCGTCT<br>GTACGGTCTGCCGCAGGATTCTTTCATCGCAGACGCAGCATGGGGCAGTCTGGGTCACGAAACCGGCTGCGT<br>TACGGGTATCGCGTATGCCAGCGATAAACGTGCAATGGCTATTGCGGGTGACGGCGGTTTTATGATGATGT<br>GCCAGTGTCTGAGCACCATTAGCCGCCATCAACTGAACTCCGTCGTGTTCGTTATTTCAAATAAAGTCTACG<br>CCATCGAACAGTCCTTTGTGGATATTTGTGCCTTCGCAAAGGGCGGTCACTTTGCGCCGTTCGATCTGCTGC<br>CGACCTGGGACTATCTGTCGCTGGCTAAAGCGTTTAGCGTGGAAGGCTACCGCGTTCAGAACGGTGAAGAAC<br>TGCTGCAAGCGCTGGAACATATCATGACCCAGAAAGATAAGCCGGCCCTGGTGGAAGTTGTCATTCAGTCGC<br>AGGATCTGGCACCGGCAATGGCTGGCCTGGTCAAAAGCATCACCGGTCACACGGTGGAACAGTGCGCCATTC<br>CGACCGGCTCGACGGAAAATCTGTATTTTCAAAGCGGTGCACTCGAG

YP_831380
ATGACGACGGTCCATGCCGCCGCCTATGAACTGCTGCGTAGCAATCGCCTGACGACGATCTTTGGTAATCCG<br>GGTGATAATGAACTGCCGTTTCTGGATGCAATGCCGGCTGACTTCCGCTATATTCTGGGCCTGCATGAGGGT<br>GTGGTTGTCGGCATGGCGGATGGTTTTGCGCAGGCCAGCGGTCAAGCGGCCTTCGTTAACCTGCATGCAGCT<br>TCTGGCACCGGTAACGCGATGGGCGCCCTGACGAATGCATGGTACAGTCACACCCCGCTGGTGATTACGGCG<br>GGCCAGCAAGTTCGTCCGATGATCGGTCTGGAAGCGATGCTGAGCAATGTTGATGCAGCCTCTCTGCCGCGC<br>CCGCTGGTCAAATGGTCTGCCGAACCGGCACAGGCTCCGGATGTTCCGCGTGCGCTGAGCCAAGCCATTCAT<br>ACCGCAACGTCTGACCCGAAGGGTCCGGTGTATCTGAGTATCCCGTACGATGACTGGAACCAGGATACCGGT<br>AATCTGTCCGAACACCTGAGCAGCCGTAGCGTGAGCCGTGCGGGTAACCCGTCAGCTGAACAACTGGATGAC<br>ATTCTGTCGGCACTGCGTGAAGCAGCTAACCCGGCGCTGGTTTTTGGTCCGGATGTGGATGCGGCCCGCGCT<br>AATCATCACGCGGTGCGTCTGGCCGAAAAACTGGCAGCTCCGGTTTGGATCGCACCGGCGGCACCGCGTTGC<br>CCGTTTCCGACCCGCCATCCGAACTTCCGTGGCGTTCTGCCGGCAAGTATTGCTGGCATCTCCGCCCTGCTGA<br>ATGGTCATGATCTGATTGTGGTTATCGGTGCACCGGTGTTCCGTTATCACCAGTACCAACCGGGCAGTTATC<br>TGCCGGAAAATTCCCGCCTGATTCACATCACCTGTGATGCAGGTGAAGCAGCTCGTGCCCCGATGGGTGATG<br>CGCTGGTTGCCGACATTGGTCAGACGCTGCGCGCGCTGGCCGACATTATCCCGCAAAGCAAACGTCCGCCGC<br>TGCGCCCGCGTGTCATCCCGCCGGTGCCGGATTCACAGGATGACCTGCTGGCACCGGACGCTGTCTTTGAAG<br>TGATGAACGAAGTCGCGCCGGAAGATGTCGTGTATGTGAATGAATCAGTTTCGACCGTCACGGCCCTGTGGG<br>AACGTGTGGAACTGAAGCATCCGGGTTCATATTACTTTCCGGCGTCGGGCGGTCTGGGTTTCGGTATGCCGG<br>CGGCCGTGGGTGTTCAGCTGGCCAACGATCGTCGCCGTGTGATTGCAGTTATCGGCGACGGTAGCGCAAATT<br>ATGGCATTACCGCTCTGTGGACGGCAGCTCAGGAAAAAATCCCGGTTGTCTTTATTATCCTGAACAATGGCA<br>CCTACGGTGCGCTGCGCGCATTCGCTAAGCTGCTGAACGCCGAAAATGCGGCCGGCCTGGATGTGCCGGGCA<br>TTTGCTTTTGTGCGATCGCCGAAGGCTATGGTGTGGAAGCGCACCGTATTACCAGCCTGGAAAACTTCAAAG<br>ATAAGCTGTCAGCAGCTCTGCAATCGGACACCCCGACGCTGCTGGAAGTGCCGACCAGCACCACGTCTCCGT<br>TTGGTAGTACGGAAAATCTGTACTTCCAGTCCGGCGCCCTCGAG
ZP_06547677
ATGAAGACCATCCACTCTGCCGCCTATGCCCTGCTGCGTCGCCACGGTATGACCACCATTTTCGGTAATCCGG<br>GTAGCAATGAACTGCCGTTTCTGAAAAGTTTCCCGGAAGACTTTCAGTATGTTCTGGGCCTGCATGAAGGTG<br>CCGTGGTTGGCATGGCAGATGGTTACGCCCTGGCAAGCGGCAAGCCGGCATTCGTGAACCTGCATGCGGCGG<br>CGGGCACCGGTAACGGCATGGGTGCCCTGACCAATTCTTGGTATAGCCACTCTCCGCTGGTGATTACGGCAG<br>GCCAGCAAGTTCGTCCGATGATCGGTGTCGAAGCGATGCTGGCCAATGTGGACGCGACCCAGCTGCCGAAAC<br>CGCTGGTTAAGTGGAGCTATGAACCGGCTAACGCGCAGGATGTTCCGCGCGCACTGTCGCAAGCTATTCATT<br>ACGCGAATACCACGCCGAAAGCCCCGGTGTATCTGAGCATCCCGTACGATGACTGGGATCAGCCGTCTGGTC<br>CGGGCGTCGAACACCTGATTGAACGTGACGTGCAAACGGCTGGCACCCCGGATGCACGTCAGCTGCAAGTTC<br>TGGTCCAGCAAGTTCAGGATGCACGTAACCCGGTGCTGGTTCTGGGTCCGGATGTGGATGCGACCCTGAGCA<br>ATGACCATGCCGTGGCACTGGCTGATAAACTGCGTATGCCGGTTTGGATCGCACCGGCTGCGAGTCGCTGCC<br>CGTTCCCGACGCGTCATCCGTCCTTTCGTGGTGTGCTGCCGGCCGCAATTGCAGGTATCAGCAAGACCCTGC<br>AAGGTCACGATCTGATTATCGTCGTGGGTGCGCCGGTTTTCCGTTATCTGCAATTTGCGCCGGGTGACTACC<br>TGCCGGTGGGTGCACAACTGCTGCATATTACGTCAGATCCGCTGGAAGCAACCCGTGCTCCGATGGGCCACG<br>CCCTGGTTGGTGATATCCGTGAAACCCTGCGCGTCCTGGCAGAAGAAGTTGTCCAGCAATCGCGCCCGTATC<br>CGGAAGCGCTGGCTGCACCGGAATGTGTGACGGACGAACCGCATCACCTGCATCCGGAAACCCTGTTCGATG<br>TCCTGGACGCAGTGGCACCGCACGATGCTATTTACGTGAAAGAAAGTACCTCCACGGTTACCGCCTTTTGGC<br>AGCGTATGAACCTGCGCCATCCGGGCAGCTATTACTTCCCGGCCGCAGGCGGTCTGGGTTTTGGTCTGCCGG<br>CTGCGGTCGGTGTGCAGCTGGCACAGCCGCAACGTCGCGTGGTTGCTCTGATTGGCGATGGTTCTGCGAACT<br>ATGGTATCACGGCACTGTGGACCGCCGCACAGTACCGTATTCCGGTCGTGTTCATTATCCTGAAAAATGGCA<br>CCTATGGTGCCCTGCGCTGGTTTGCAGGTGTCCTGAAGGCTGAAGATAGTCCGGGCCTGGACGTGCCGGGTC<br>TGGATTTCTGCGCAATCGCTAAAGGCTACGGTGTTAAGGCGGTCCATACGGATACCCGTGACTCCTTTGAAG<br>CTGCACTGCGTACGGCGCTGGATGCAAACGAACCGACCGTGATTGAAGTTCCGACGCTGACCATCCAGCCGC<br>ACGGCTCAACCGAAAATCTGTATTTTCAATCGGGTGCGCTCGAG
1OVM_P23234

ATGCGTACCCCGTACTGCGTTGCTGACTACCTGCTGGACCGTCTGACCGATTGCGGCGCGGACCACCTGTTT<br>GGCGTGCCGGGCGACTACAACCTGCAATTTCTGGACCATGTCATTGATTCTCCGGACATCTGCTGGGTGGGC<br>TGTGCCAACGAACTGAATGCAAGTTATGCGGCCGATGGCTACGCACGTTGCAAAGGTTTTGCAGCTCTGCTG<br>ACCACGTTCGGCGTGGGTGAACTGTCCGCGATGAATGGCATTGCCGGCAGCTATGCGGAACATGTGCCGGTT<br>CTGCACATCGTTGGCGCGCCGGGCACCGCGGCGCAGCAACGTGGTGAACTGCTGCATCACACGCTGGGCGAT<br>GGTGAATTTCGCCATTTCTACCACATGTCCGAACCGATTACCGTTGCCCAAGCAGTCCTGACGGAACAGAAC<br>GCCTGCTATGAAATCGACCGTGTGCTGACCACGATGCTGCGCGAACGTCGTCCGGGCTATCTGATGCTGCCG<br>GCTGATGTTGCGAAAAAGGCAGCTACCCCGCCGGTCAACGCACTGACGCATAAACAGGCTCACGCGGATTCC<br>GCTTGTCTGAAGGCGTTTCGTGACGCGGCCGAAAATAAACTGGCCATGTCAAAGCGTACCGCCCTGCTGGCA<br>GACTTCCTGGTGCTGCGTCATGGCCTGAAACACGCGCTGCAAAAATGGGTTAAGGAAGTCCCGATGGCCCAT<br>GCAACCATGCTGATGGGCAAGGGTATTTTTGATGAACGCCAGGCCGGCTTCTATGGCACCTACTCAGGCTCG<br>GCCAGCACGGGTGCAGTGAAAGAAGCTATCGAAGGCGCGGATACCGTGCTGTGCGTTGGTACGCGTTTTACC<br>GACACGCTGACCGCCGGTTTCACGCATCAGCTGACCCCGGCACAAACGATTGAAGTTCAGCCGCACGCAGCT<br>CGCGTCGGTGATGTGTGGTTTACCGGTATTCCGATGAACCAAGCGATCGAAACGCTGGTTGAACTGTGTAAA<br>CAGCATGTCCACGCTGGCCTGATGAGCAGCAGCAGCGGTGCCATTCCGTTCCCGCAACCGGATGGCTCTCTG<br>ACCCAGGAAAATTTTTGGCGTACGCTGCAAACCTTCATTCGTCCGGGCGATATTATCCTGGCGGACCAGGGC<br>ACCTCTGCTTTTGGTGCGATCGATCTGCGTCTGCCGGCCGACGTGAACTTCATTGTTCAACCGCTGTGGGGC<br>AGTATCGGTTATACCCTGGCGGCGGCGTTTGGCGCCCAGACGGCATGTCCGAATCGTCGCGTCATTGTGCTG<br>ACCGGCGATGGTGCTGCGCAGCTGACGATCCAAGAACTGGGTAGCATGCTGCGCGACAAACAACATCCGATT<br>ATCCTGGTGCTGAACAATGAAGGCTATACCGTTGAACGTGCCATTCATGGTGCAGAACAGCGCTACAACGAT<br>ATTGCACTGTGGAATTGGACCCACATCCCGCAAGCGCTGTCTCTGGACCCGCAGAGTGAATGCTGGCGTGTG<br>TCGGAAGCTGAACAGCTGGCGGATGTCCTGGAAAAAGTGGCGCATCACGAACGCCTGAGCCTGATTGAAGTT<br>ATGCTGCCGAAAGCTGATATCCCGCCGCTGCTGGGTGCGCTGACCAAGGCTCTGGAAGCGTGTAACAATGCC<br>GGCTCGACCGAAAATCTGTATTTTCAGAGCGGTGCACTCGAG
ZP_06418208
ATGGGCGCACATTACCCGATGAGCGGCGAATCCACCGTCCATGATGTCACCTACCAACTGCTGCGTTCACTG<br>GGCATTACGACCGTTTTTGGCAATCCGGGCAGCACCGAACAGACGTTTCTGCAAGATTTCCCGTCGGACTTT<br>ACCTATGTCCTGGGTCTGCAAGAAGCCTCCGTGATGGCAATGGCTGATGCATTCGCACAGGTTACGCGTCGC<br>CCGGCCCTGGTCAACCTGCATAGCAGCGCGGGCGTTGGTCACTCAATTGGCAATCTGGTCTCGGCTTTTGAC<br>GCGCATACCCCGCTGATTGTGACGGCGGGTCAGCAACACCGTGAAATGGTTATCGGCGAACCGGCCCTGTCA<br>AACCGTGAAGCAACCAATCTGCCGCGCCCGTGGGTCAAATGGTCGTACGAACCGGCACGTGCTCAGGATGTG<br>CCGGAAGCATTCATGCGTGCATGCGCAATTGCAACCCAGCCGCCGGCGGGTCCGGTTTTTCTGTCTATCCCG<br>CTGGATGACTGGAACGCTCCGATGACCGGTCCGGCGGTGGTTCGCAGTGTTTCCACCGTCTGTGCGCCGGAA<br>ACGGAACGTCTGCGCGGTTTCGCTCGTCGCATTCGTGCGAGTCAGCGCCCGGTTCTGGTCTTTGGCCCGGAA<br>GTGGATCGTTCCGGCGGTTGGCATGCAGCAATCGCTCTGGCGGAAAATCTGGGTGTGCCGGTTTTCGGCGCA<br>GCTGGTCCGGATCGCGTTAGCTTCCCGGAAGACCACCCGCTGTTTCAAGGCCGTCTGGGCATGTCTCAGAAG<br>TCAGTGTCGGATCGCCTGACCGGTTATGACCTGGTCGTGGTTATTGGCGCGGCCGTGTTTCGTTATTACCCG<br>CATGTTCCGGGTGATATTCTGCCGGCCGGCACCGAACTGCTGCACATCACGGGCGACCCGGCCGTGGCAGGT<br>GCAGCTCGTGTTGGTGATAGTGTCCTGGGTGACGCACGCCTGGCAATCGAACTGCTGACCGAACTGCTGGAT<br>GCAGAAGCAGCACATAGCCCGCGTCGCCCGCAGCATCAACACCCGCATCCGCAGCCGCAACACGTGGAACAT<br>GTTCAGCACCAGCAACCGCCGCAGACCCGTTTTCAAGCTCGCGCGCAGCCGCGTCCGCCGCGTCATGCACCGG<br>ATCGTCCGGAAACGGCCGGCGGTCCGCTGTGCGCAGACGAAGTCCACGCTGTGATTAACGCGAGCCGTCCGC<br>GCAATGCAGCTCTGGTTTATGAAAGCACCTCTACGATTGGTGAACAGGTCGAATGGCTGCCGGTGATCGAAC<br>CGGCCAGCTTTTTCGCAAACGCATCTGGCGGTCTGGGTTGGGCGGTGCCGGCGGCCGTCGGTGTGGCACTGG<br>GTGATCGTGACCGCGGTGTGCATCGCCCGGTTATCGGCATTATCGGCGATGGTGCGTTCCAATACAGCGTTC<br>AGGCCCTGTGGACCGCAACGCAGCATTCTCTGCCGATTGTGTTCGTCGTGCTGCGTCACGAATATAGTATCC<br>TGAAATCCTTTGCTGAACTGGAACGCACCGCGGGCGTGGGTAGTACGGAAAATCTGTACTTTCAGTCCGGTG<br>CGCTCGAG
ZP_07290467
ATGCGTACGGTGCGTGAATCGGCTCTGGACGTGCTGCGTGCGCGTGGTATGACGACGGTTTTTGGTAATCCG<br>GGCTCAACGGAACTGCCGATGCTGAAACAGTTTCCGGATGACTTCCGCTATGTTCTGGGTCTGCAAGAAGCT<br>GTGGTTGTCGGTATGGCAGATGGCTTTGCCCTGGCAAGTGGCACCACGGGTCTGGTGAATCTGCATACCGGT<br>CCGGGCACGGGTAACGCGATGGGCGCAATTCTGAACGCTCGTGCGAATCGTACCCCGATGGTGGTTACGGCG<br>GGCCAGCAAGTGCGTGCCATGCTGACGATGGAAGCACTGCTGACCAATCCGCAGAGTACGCTGCTGCCGCAA

CCGGCTGTCAAGTGGGCGTACGAACCGCCGCGCGGCCGATGTGGCACCGGCACTGGCTCGTGCGGTCCAG<br>GTGGCAGAAACCCCGCCGCAAGGTCCGGTTTTTGTCTCCCTGCCGATGGATGACTTCGATGTCGTGCTGGGC<br>GAAGATGAAGACCGTGCAGCTCAGCGTGCGGCGGCACGTACCGTTACGCACGCTGCGGCCCCGAGCGCGGAA<br>GTTGTCCGTCGCCTGGCAGCTCGTCTGAGTGGTGCTCGTTCCGCGGTGCTGGTTGCGGGTAATGATGTGGAC<br>GCCTCTGGCGCATGGGATGCTGTGGTTGAACTGGCCGAACGTACCGGTCTGCCGGTCTGGAGTGCACCGACG<br>GAAGGTCGTGTGGCATTTCCGAAATCCCATCCGCAGTATCGTGGTATGCTGCCGCCGGCAATTGCACCGCTG<br>AGCCGTTGCCTGGAAGGTCACGATCTGGTCCTGGTGATCGGTGCGCCGGTGTTCTGTTATTACCCGTACGTT<br>CCGGGTGCCCATCTGCCGGAAAACACCGAACTGGTTCACCTGACGCGCGATGCAGACGAAGCAGCCCGTGCC<br>CCGGTTGGTGATGCAGTCGTGGCCGACCTGGCACTGACCGTGCGCGCTCTGCTGGCGGAACTGCCGGCGCGT<br>GAAGCAGCTGCGCCGGCCGCACGTACCGCTCGCGCGGAATCTACGGCCGAAGTCGATGGTGTGCTGACCCCG<br>CTGGCTGCAATGACGGCAATTGCACAGGGCGCTCCGGCAAACACCCTGTGGGTTAATGAAAGCCCGTCTAAC<br>CTGGGTCAATTTCATGATGCAACCCGTATCGACACGCCGGGCAGCTTTCTGTTCACCGCCGGCGGTGGCCTG<br>GGTTTCGGTCTGGCCGCAGCTGTGGGTGCCCAGCTGGGCGCACCGGATCGTCCGGTTGTCTGCGTTATTGGC<br>GACGGTTCAACCCACTATGCAGTCCAGGCACTGTGGACCGCGGCGGCGTACAAAGTTCCGGTCACCTTTGTG<br>GTTCTGTCGAATCAGCGCTATGCAATCCTGCAATGGTTCGCGCAAGTGGAAGGCGCTCAAGGTGCGCCGGGC<br>CTGGATATTCCGGGTCTGGACATCGCTGCGGTTGCAACGGGTTACGGTGTCCGTGCCCATCGTGCAACCGGC<br>TTTGGTGAACTGTCAAAGCTGGTGCGTGAATCGGCGCTGCAACAAGATGGCCCGGTTCTGATCGACGTGCCG<br>GTTACCACGGAACTGCCGACCCTGGGTAGCACGGAAAACCTGTATTTCCAGTCTGGCGCGCTCGAG
Native KIVD
ATGTATACAGTAGGAGATTACCTATTAGACCGATTACACGAGTTAGGAATTGAAGAAATTTTTGGAGTCCC<br>TGGAGACTATAACTTACAATTTTTAGATCAAATTATTTCCCGCAAGGATATGAAATGGGTCGGAAATGCTA<br>ATGAATTAAATGCTTCATATATGGCTGATGGCTATGCTCGTACTAAAAAAGCTGCCGCATTTCTTACAACCT<br>TTGGAGTAGGTGAATTGAGTGCAGTTAATGGATTAGCAGGAAGTTACGCCGAAAATTTACCAGTAGTAGAA<br>ATAGTGGGATCACCTACATCAAAAGTTCAAAATGAAGGAAAATTTGTTCATCATACGCTGGCTGACGGTGA<br>TTTTAAACACTTTATGAAAATGCACGAACCTGTTACAGCAGCTCGAACTTTACTGACAGCAGAAAATGCAA<br>CCGTTGAAATTGACCGAGTACTTTCTGCACTATTAAAAGAAAGAAAACCTGTCTATATCAACTTACCAGTTG<br>ATGTTGCTGCTGCAAAAGCAGAGAAACCCTCACTCCCTTTGAAAAAGAAAACTCAACTTCAAATACAAGT<br>GACCAAGAGATCTTGAACAAAATTCAAGAAAGCTTGAAAAATGCCAAAAAACCAATCGTGATTACAGGACA<br>TGAAATAATTAGTTTTGGCTTAGAAAAAACAGTCTCTCAATTTATTTCAAAGACAAAACTACCTATTACGA<br>CATTAAACTTTGGAAAAAGTTCAGTTGATGAAGCTCTCCCTTCATTTTTAGGAATCTATAATGGTAAACTC<br>TCAGAGCCTAATCTTAAAGAATTCGTGGAATCAGCCGACTTCATCCTGATGCTTGGAGTTAAACTCACAGAC<br>TCTTCAACAGGAGCCTTCACTCATCATTTAAATGAAAATAAAATGATTTCACTGAATATAGATGAAGGAAA<br>AATATTTAACGAAAGCATCCAAAATTTTGATTTTGAATCCCTCATCTCCTCTCTCTTAGACCTAAGCGAAAT<br>AGAATACAAAGGAAAATATATCGATAAAAAGCAAGAAGACTTTGTTCCATCAAATGCGCTTTTATCACAAG<br>ACCGCCTATGGCAAGCAGTTGAAAACCTAACTCAAAGCAATGAAACAATCGTTGCTGAACAAGGGACATCA<br>TTCTTTGGCGCTTCATCAATTTTCTTAAAACCAAAGAGTCATTTTATTGGTCAACCCTTATGGGGATCAATT<br>GGATATACATTCCCAGCAGCATTAGGAAGCCAAATTGCAGATAAAGAAAGCAGACACCTTTTATTTATTGG<br>TGATGGTTCACTTCAACTTACGGTGCAAGAATTAGGATTAGCAATCAGAGAAAAAATTAATCCAATTTGCT<br>TTATTATCAATAATGATGGTTATACAGTCGAAAGAGAAATTCATGGACCAAATCAAAGCTACAATGATATT<br>CCAATGTGGAATTACTCAAAATTACCAGAATCATTTGGAGCAACAGAAGAACGAGTAGTCTCGAAAATCGT<br>TAGAACTGAAAATGAATTTGTGTCTGTCATGAAAGAAGCTCAAGCAGATCCAAATAGAATGTACTGGATTG<br>AGTTAATTTTGGCAAAAGAAGATGCACCAAAAGTACTGAAAAAAATGGGCAAACTATTTGCTGAACAAAAT<br>AAATCATAA
CAK95977
ATGAAGACGGTCCACGGTGCAACCTACGACATCCTGCGCCAGCATGGTCTGACGACGATTTTTGGTAATCCG<br>GGTGATAACGAACTGCCGTTTCTGAAAGGTTTCCCGGAAGACTTTCGTTATATTCTGGGCCTGCATGAAGGT<br>GCCGTGGTTGGCATGGCAGATGGTTACGCGCTGGCCAGTGGTCAGCCGACCTTTGTGAACCTGCATGCGGCG<br>GCGGGCACCGGTAACGGCATGGGTGCACTGACGAATGCTTGGTATAGTCACTCCCCGCTGGTTATTACGGCG<br>GGTCAGCAAGTCCGCTCTATGATCGGCGTGGAAGCTATGCTGGCGAACGTGGACGCTGCACAGCTGCCGAAA<br>CCGCTGGTTAAGTGGTCACATGAACCGGCAACCGCTCAGGATGTGCCGCGTGCGCTGTCGCAAGCCATTCAC<br>ACGGCAAATCTGCCGCCGCGCGGTCCGGTGTATGTTTCAATCCCGTACGATGACTGGGCCTGCGAAGCACCG<br>TCGGGTGTTGAACATCTGGCGCGTCGCCAGGTCAGCTCTGCCGGCCTGCCGAGCCCGGCACAGCTGCAACAC<br>CTGTGTGAACGTCTGGCCGCAGCTCGTAACCCGGTCCTGGTGCTGGGTCCGGATGTGGATGGTTCTGCGGCC<br>AATGGCCTGGCTGTTCAGCTGGCGGAAAAGCTGCGTATGCCGGCTTGGGTGGCACCGTCAGCCTCGCGCTGC

CCGTTCCCGACCCGTCACGCCTGTTTTCGCGGTGTTCTGCCGGCAGCTATTGCCGGTATCAGCCATAACCTGG<br>CAGGCCACGATCTGATTCTGGTCGTGGGTGCGCCGGTGTTCCGTTATCATCAGTTTGCGCCGGGTAATTACC<br>TGCCGGCGGGTTGCGAACTGCTGCACCTGACCTGTGATCCGGGTGAAGCAGCCCGCGCTCCGATGGGTGACG<br>CGCTGGTTGGCGATATCGCCCTGACCCTGGAAGCAGTGCTGGATGGCGTTCCGCAGAGCGTCCGTCAAATGC<br>CGACGGCACTGCCGGCAGCTGAACCGGTGGCAGATGACGGTGGTCTGCTGCGTCCGGAAACCGTTTTCGACC<br>TGCTGAACGCGCTGGCCCCGAAAGATGCCATTTATGTTAAGGAAAGCACCTCTACGGTCGGTGCATTCTGGC<br>GTCGCGTGGAAATGCGTGAACCGGGCTCCTACTTTTTCCCGGCGGCCGGCGGTCTGGGTTTTGGTCTGCCGG<br>CAGCTGTTGGTGTCCAGCTGGCCAGTCCGGGTCGCCAAGTGATTGGCGTTATCGGCGATGGTTCCGCTAACT<br>ATGGTATTACCGCACTGTGGACGGCGGCCCAGTACAACATCCCGGTTGTCTTCATTATCCTGAAAAATGGCA<br>CCTATGGTGCTCTGCGTTGGTTTGCGGATGTCCTGGACGTGAATGATGCGCCGGGTCTGGACGTGCCGGGCC<br>TGGATTTCTGCGCAATCGCTCGCGGCTACGGTGTTCAGGCAGTCCATGCAGCTACCGGCAGCGCATTTGCCC<br>AAGCACTGCGTGAAGCGCTGGAATCTGATCGCCCGGTGCTGATTGAAGTTCCGACCCAGACGATCGAACCGG<br>GTAGTACGGAAAATCTGTATTTTCAATCCGGCGCGCTCGAG
ZP_07282849
ATGGCTCGCTTTGGTGTGCGTAAGGCTTTTGGTGTGGTTGGTAGCGGTAACTTTCACTTCACGAATGGTCTG<br>ATTCAAGGTGGTGCCGAATTTGTGGCAGCACGTCATGAAGGCGGTGCAACCACGATGGCAGATGCTTACGCG<br>CGTTGCTCAGGCGAAGTTGCAGCTGTCTCGGTGCACCAGGGTTGTGGTCTGGGTAACGCAACCACGGGTATT<br>GGCGAAGCGGCCAAAAGTCGCACCCCGCTGGTTGTGGTCACCGCCGAAGCAACCGATCCGCTGTCCAATTTC<br>CATATTGATCAGGACGCTCTGGCGCGCAGTGTTGGTGCGAGCAGCATCCTGGTCCGTTCCGCAAAGACCGCC<br>CTGGCAGATGTGCGTCGCGCGTTTACCCAGGCACGTCAAGACCGTCGCACGGTGCTGCTGCGTCTGCCGCTG<br>GAAGTTCAGGCGGAACCGTTCGATGAAAGCCTGCTGGAAGGTCTGACCGCAATTGAAGCTCTGCCGCAACCG<br>CGCGCAGCTGAAGCAGAAGTTCAGGCTCTGGCGTCAATCCTGCAACGTGCGGAACGCCCGGTCTTTCTGTGC<br>GGTCGTGGCTCGCGCGCGGCCCGTGCGGAACTGGTTGCCCTGGCAGATCGTTGTGGCGCGCTGCTGGCCGAA<br>GGTGCTGTGGCAAAAGGTCTGTTTGCAGGCGAACCGTGGGCTATTGGCGTTAGCGGCGGTTTCAGTTCCCCG<br>CTGACCACGGAACTGATCCAGGGTGCGGATGTGGTTGTCGGTTGGGGCTCTGCGCTGAACGACTGGACCACG<br>GCGCATGGCCGTCTGCTGAGCCCGGAAACCACGCTGGTTCAAGTCGATCTGGAAAGCGCGGCGCTGGGTCGT<br>AATCGTCCGGTGGATCTGGGTATTGTCGGTGACGTGGGCGGCACCGCCCTGGCAGTCGCTGAACTGCTGGAA<br>GTGCACAACGGCTATCGTAGCGCGGAACTGAAGACGCGCATTGCGCGTGAAATCCGTTGGCGCGATAATGAA<br>TATGATGACGTGTCAACCGGTGAAGTTATCGATCCGCGTACGCTGTCGGCGGCCCTGGACGAACTGCTGCCG<br>GCGAACCGTGTGGTTGGTGTGGATAGTGGCAATTTTATGGGTTATCCGACCATGTACCTGGATGTTCCGGAC<br>GAAAACGGCTTTTGCTTCACGCAGGCGTTCGCCTCCATTGGTCTGGGCCTGGCAACCGCTATCGGCACGGCG<br>CTGGCCCGTCCGGATCGTTTTCCGGTGGCAGCTTGTGGTGACGGCGGTTTCCTGATGAGTATTGCGGAACTG<br>GAAACCGTCGTGCGTCTGAAACTGCCGATGCTGATCGTTGTCTATAATGATCATGCATACGGTGCTGAAGTC<br>TATTTCTTTGAACCGGGCGGTCACCCGGCCGACACCGTGACGTTTCCGGATACCGACCTGGCAGCAATTGCC<br>CGCGGTTACGGTTGCGATGCAGTGACCGTTCGTACGAAAGAAGACCTGGCAGAAGTTGCAACCCGCGTCGCA<br>GCTGGCCTGGATCGTCCGCTGGTTGTGGATGCAAAGATCGCTGGTTTTAGCGCATGGTGGCTGCAAGCAGCA<br>ATGACCCATCACGGCAGCACGGAAAACCTGTATTTCCAATCTGGTGCACTCGAG
ZP_06846103
ATGACCAGCCGTAGCTCGTTTAGCCCGCCGTCAGCGTCAGAACAGCGTGGTGCGGATATTTTTGCCGAAGTC<br>CTGCAATGTGAAGGTGTCCGCTATATTTTTGGCAATCCGGGCACCACGGAACTGCCGCTGCTGGATGCACTG<br>ACCGACATTACGGGTATCCATTATGTGCTGGGCCTGCACGAAGCGTCAGTGGTTGCGATGGCCGATGGTTAC<br>GCACAGGCTTCGGGCAAACCGGGTTTCGTTAACCTGCATACCGCCGGCGGTCTGGGTAATGCGATGGGTGCC<br>ATTCTGAACGCAAAGATGGCTAATACCCCGCTGGTCGTGACGGCGGGTCAGCAAGATACCCGTCATGGCGTT<br>ACCGATCCGCTGCTGCACGGCGACCTGACCGGTATCGCACGTCCGAATGTCAAATGGGCCGAAGAAATTCAT<br>CACCCGGAACATATCCCGATGCTGCTGCGTCGTGCGCTGCAAGATTGCCGCACGGGTCCGGCTGGTCCGGTG<br>TTTCTGAGTCTGCCGATTGACACGATGGAACGTTGTACGTCCGTGGGTGCAGGTGAAGCCAGCCGTATCGAA<br>CGCGCGAGCGTGGCTAACATGCTGCATGCGCTGGCCACCGCACTGGCTGAAGTGACGGCCGGTCACATTGCG<br>CTGGTCGCCGGTGAAGAAGTGTTCACCGCGAATGCCAGTGTTGAAGCAGTCGCTCTGGCGGAAGCACTGGGC<br>GCACCGGTTTTTGGTGCTTCCTGGCCGGGTCATATTCCGTTCCCGACCGCACACCCGCAGTGGCAGGGTACGC<br>TGCCGCCGAAGGCGAGCGATATCCGTGAAACCCTGGGCCCGTTTGACGCCGTGCTGATTCTGGGCGGTCATA<br>GTCTGATCTCCTATCCGTACTCAGAAGGTCCGGCAATTCCGCCGCACTGCCGCCTGTTCCAGCTGACCGGCGA<br>TGGTCATCAAATCGGCCGTGTTCACGAAACCACGCTGGGCCTGGTGGGCGATCTGCAACTGAGTCTGCGCGC<br>GCTGCTGCCGCTGCTGGCCCGTAAACTGCAACCGCAAAACGGTGCAGTCGCTCGTCTGCGCCAAGTGGCAAC<br>CCTGAAGCGTGATGCTCGTCGCACGGAAGCGGCCGAACGTTCAGCCCGCGAATTTGACGCGTCGGCCACCAC

GCCGTTTGTTGCAGCTTTCGAAACCATTCGCGCAATCGGCCCGGATGTGCCGATTGTTGACGAAGCGCCGGT<br>TACGATCCCGCATGTCCGTGCCTGCCTGGATAGCGCATCTGCTCGCCAGTACCTGTTTACCCGTTCTGCAATT<br>CTGGGTTGGGGTATGCCGGCGGCCGTCGGTGTGAGTCTGGGTCTGGATCGTTCCCCGGTTGTCTGTCTGGTG<br>GGCGACGGTTCAGCGATGTACTCGCCGCAGGCACTGTGGACCGCAGCTCACGAACGCCTGCCGGTTACGTTT<br>GTGGTTTTCAACAATGGTGAATATAACGCCCTGAAAAATTTTGCGCGTGCCCAAACCAACTACCGTAGCGCA<br>CGCGCTAATCGTTTTATTGGCCTGGATATCTCTGACCCGGCGATTGATTTCCCGGCGCTGGCCAGCTCTCTG<br>GGTGTGCCGGCACGTCGCGTTGAACGTGCTGGTGATATTGCAATCGCTGTCGAAGACGGCATCCGCAGCGGT<br>CGTCCGAACCTGATTGATGTGCTGATCAGTTCCTCATCGGGTAGCACCGAAAATCTGTACTTTCAGTCTGGC<br>GCGCTCGAG
ZP_04996569
ATGCTGCGTACGGCGGGTGAAGAATCTGGCGTCAAGGTGCGCGATGCGTTTTTCGAAGTGCTGCGTTCCCAC<br>GGCATTACCACGGTTTTTGGCAATCCGGGCAGCAATGAACTGCCGCTGCTGCGCGATTTTCCGGATGACTTC<br>CGTTATGTGCTGGCGCTGCATGAGGGTGCGGCCATTGCTATGGCGGATGGTTACGCCCTGGCAACCGGCCGT<br>CCGTCGCTGGTCAACCTGCATGCAGCTGCGGGCACGGGTAATGCGATGGGCAACCTGACCAATACGCAGTCA<br>GGTCACGTGCCGGTGGTTGTCACCTCGGGTCAGCAAGCACGTCGCTATACGGCACTGAACGCCCTGCTGACC<br>AATGTTGATGCTACGGCGCTGGCCGAACCGCTGGTCAAATGGTCATGCGAACCGCTGCGTCCGGAAGACGTG<br>CCGCAGGCACTGAGTCAAGGCATTCTGCTGGCAGGTTCCGCACCGGCTGGCCCGGTTTACCTGAGCCTGCCG<br>CTGGATGACTGGGATCATCAGGCCGATCCGGGCGCGCTGAAACACCTGAAGGCCCGTACCGTTCAAGGCGAT<br>CCGGTGGTTTCCGAACCGGCACTGGACCTGCTGCGTCGCCGTCTGACCGGTGCAGCAAACCCGGTGATGGTC<br>GTGGGTCCGGGCATCGATGACGCAACGGGTTGGGATGGTGCATGCCGCCTGGCTGACCGTCTGGCGCTGCCG<br>GTTTTTGTCGCACCGAGCCCGTCTCGCTGTCCGTTCCCGACCCGTCATCCGGGCTATCGTGGTGTGCTGCCGT<br>CTGATATTCCGGCGGTTGCCCGCCATTTTGATGGTCACGACCTGGTTGTCGCGTTTGGCGCTGCGATCTTCC<br>GTTATTTTGCCTTCGAAGAAGGTGATTATCTGCCGCCGGGCACCGAACTGTGGGCAGTTACGAGCGATCCGG<br>ACGAAGCAACCCGCGCTCCGTTTGGTCGTATTCTGGTCGGCAACCCGTCTGATGCACTGGCTCGTCTGACCG<br>AAACGGTGCCGGCACGTCATCGTCCGCCGCCGCCGCCGCTGGAACGCACCAGTCGTCTGAATGAAGCAGGTC<br>CGGCATTCTCCGCGGAAGCAATCGTGGATGCACTGGACGCCGCAAAGGATGAAAGCACCGTCCTGGCGCACG<br>AATGGACGTCTGTGCTGACCACGTGGGATCGCTTTGACATTTCACGTCCGGGCTCGCTGTATTTCCCGGCCA<br>GTGGCGGTCTGGGTTGGGGTCTGCCGGCTGCGATCGGTCTGCAACTGGGTGATCCGTCACGCCGTGTTCTGG<br>CCATGCTGGGCGACGGTGCACTGCATTATACCGTGTCGGCACTGTGGACGGCCGCACGCTACCGTGTTCCGG<br>TGGTTTTTGTCGTGGCTCGCAACGGCGAATATGGTGCGCTGAAAAAGTTCACCCAGGCAATGCAAGCTCCGG<br>GCGTGCCGGGTCTGGAACTGCCGGGTATTGATATCACCGGCATTGCAAGCGCTTACGGCATTTCTGCTACCC<br>GTATCGATACGCTGGACGCACTGACCGCTGCGGTTACGGCCGCACTGGCGACCGATGAACCGCACCTGATCG<br>AAGTCCCGCAGCAACCGCTGACCGCGAGTGGTTCCACGGAAAATCTGTACTTTCAGAGCGGCGCCCTCGAG
YP_381143
ATGGCCCCGTACCGTGCCCCGCACCGTAGCCCGCACTCGTACTTTACCATGAAGGGTCACGAAGCGATTCTG<br>CGTCAGTTTCTGGCGAACGGCATGGATCACATGTTTGGCACGCCGGGCGACGTCGAACAAGGTTTCCTGGAT<br>GCGCTGGCCGACGTGCCGGAAATGAAATATATTCTGACGCTGCAAGAAAGCATCGCTGTGCTGTGTGCGGAT<br>GGTTACGCACGTGCTCGCCTGAAGCCGGCGCTGGTTCAGATTCACAGCTCTCCGGGCCTGGGTAACGCCATC<br>GGCAATCTGTATCAGGCTATGCGTGGTCAAGCGCCGCTGGTTGTGATTGGCGGTGATGCGGGTATCAAATAC<br>CAGGCAATGGATGCTCAAATGGCGGCCGACCTGGTGGCGATGGCCGAACCGGTTACCAAATGGAGCGCGATG<br>GTCCAGCATCCGAGTTCCCTGCTGCGCATGGTCCGTCGCGCCATTAAGGTGGCAGCTACGCCGCCGTGCGGTC<br>CGGTTTATCTGTGTCTGCCGGAAGATATTCTGGACGCGGAAATCACCGAAAAAATTATCCCGGCCCACATTC<br>CGTCCCTGGAAACGTGTCCGGGTTCACTGGATCTGGACCGTATGGTGTCTGCCATCCAGAGTGCACAAAACC<br>CGATTATCCTGGTCGGTGATGGTGTGGCATGGACCGGCGGTGTTGAAAAAATTGTGGATCTGGCCGAAACG<br>CTGGGCGCAAAGGTTTATTCTGCTGATGGCGGTGAAATCAACTTTCCGGATGACCATCTGCTGAATTACGGC<br>AGTACCGGTGCCATGTTCGGTGATCAGTCTCTGCCGATTATGCAAAGTTGCGACCTGTGTCTGACGCTGGGC<br>TGCTATCTGCTGCCGGAAGTGTTTCCGCATCTGGGTGATATCTTCAACGAAGACGCAACCATTATCCACGTG<br>GATACGAACGTTGACAATATTGCAAAAAATCATCGCGTTGATATCTCGTACGTCGCTGAACCGCACAGCGTC<br>GTGACCGGTCTGCTGCCGATTCTGAAATCGCTGTCATCGAGCTGGCATAATGCGGCCCAGCAACGTCGCAGC<br>AAGCTGGAATCTGAAAGTCCGGTTGTCCACAACAATGTTGACCAGAACTATCAAGTCGAACCGCCGTATCCG<br>TCTGAAGCGTACGATGGCATTAATCGTTCCATGCGCTCAGGTTACTTTATCAAAACCCTGGCAGATAAACTG<br>CCGAAGGAAACCATTATCTTCGACGAAGCTCTGACGAACTCCCCGCCGGTGAATCGTTATCTGCCGGGCCAG<br>AAACCGGGTGATCGTATGCTGACCCGCGGCGGTTCACTGGGCACGGGTTTTCCGGGCGCGATTGGTGCCAAG

```
ATCGCATATCCGGATCGCTGCGTTATTGGCTTCTCGGGTGACGGCGGTAGCATGTACACCATCCAGTGTCTG
TGGACGGCCGTCCGTCATAACGTGGCAGCTAAATTTATTGTGTGCCAGAACCGCAGTTATAAACTGCTGCAA
TCGAATATTAGCAAGTTCTGGCAGGAACGTGGCATCGAAGGTCGCGAATTTCCGGTTCCGTTCGATCTGTCT
AAACCGGAAATTTGTTTTAGTGTGATCGCAAATTCCTTCGGCGTTTCAGGTGAACGTGTGGTTCGCCCGGAT
CAAGTGGGCGAAGCGATTGATCGTATGCTGAACCACGACGGTCCGTATCTGATCAATCTGGTTCTGGATGGC
GACATTCGTCCGGATCTGATCGGTGTCCGCTGCGGCCAAGGTTCCACCGAAAACCTGTACTTTCAGTCAGGT
GCGCTCGAG
```

Supplementary Table 4. Protein sequences for all genes used in this study.

Protein Sequences
3FZN
MASVHGTTYELLRRQGIDTVFGNPGSNELPFLKDFPEDFRYILALQEACVVGIADGYAQASRKPAFINLHSAA<br>GTGNAMGALSNAWNSHSPLIVTAGQQTRAMIGVEALLTNVDAANLPRPLVKWSYEPASAAEVPHAMSRAIH<br>MASMAPQGPVYLSVPYDDWDKDADPQSHHLFDRHVSSSVRLNDQDLDILVKALNSASNPAIVLGPDVDAAN<br>ANADCVMLAERLKAPVWVAPSAPRCPFPTRHPCFRGLMPAGIAAISQLLEGHDVVLVIGAPVFRYHQYDPGQ<br>YLKPGTRLISVTCDPLEAARAPMGDAIVADIGAMASALANLVEESSRQLPTAAPEPAKVDQDAGRLHPETVFD<br>TLNDMAPENAIYLNESTSTTAQMWQRLNMRNPGSYYFCAAGGLGFALPAAIGVQLAEPERQVIAVIGDGSAN<br>YSISALWTAAQYNIPTIFVIMNNGTYGALRWFAGVLEAENVPGLDVPGIDFRALAKGYGVQALKADNLEQLK<br>GSLQEALSAKGPVLIEVSTVSPVKGSTENLYFQSGALE
2VBI
MTYTVGMYLAERLVQIGLKHHFAVAGDYNLVLLDQLLLNKDMKQIYCCNELNCGFSAEGYARSNGAAAAVV<br>TFSVGAISAMNALGGAYAENLPVILISGAPNSNDQGTGHILHHTIGKTDYSYQLEMARQVTCAAESITDAHSAP<br>AKIDHVIRTALRERKPAYLDIACNIASEPCVRPGPVSSLLSEPEIDHTSLKAAVDATVALLEKSASPVMLLGSKL<br>RAANALAATETLADKLQCAVTIMAAAKGFFPEDHAGFRGLYWGEVSNPGVQELVETSDALLCIAPVFNDYST<br>VGWSAWPKGPNVILAEPDRVTVDGRAYDGFTLRAFLQALAEKAPARPASAQKSSVPTCSLTATSDEAGLTND<br>EIVRHINALLTSNTTLVAETGDSWFNAMRMTLPRGARVELEMQWGHIGWSVPSAFGNAMGSQDRQHVVM<br>VGDGSFQLTAQEVAQMVRYELPVIIFLINNRGYVIEIAIHDGPYNYIKNWDYAGLMEVFNAGEGHGLGLKATT<br>PKELTEAIARAKANTRGPTLIECQIDRTDCTDMLVQWGRKVASTNARKTTLAGSTENLYFQSGALE
1ZPD
MSYTVGTYLAERLVQIGLKHHFAVAGDYNLVLLDNLLLNKNMEQVYCCNELNCGFSAEGYARAKGAAAAVV<br>TYSVGALSAFDAIGGAYAENLPVILISGAPNNNDHAAGHVLHHALGKTDYHYQLEMAKNITAAAEAIYTPEEA<br>PAKIDHVIKTALREKKPVYLEIACNIASMPCAAPGPASALFNDEASDEASLNAAVDETLKFIANRDKVAVLVGS<br>KLRAAGAEEAAVKFTDALGGAVATMAAAKSFFPEENALYIGTSWGEVSYPGVEKTMKEADAVIALAPVFNDY<br>STTGWTDIPDPKKLVLAEPRSVVVNGIRFPSVHLKDYLTRLAQKVSKKTGSLDFFKSLNAGELKKAAPADPSA<br>PLVNAEIARQVEALLTPNTTVIAETGDSWFNAQRMKLPNGARVEYEMQWGHIGWSVPAAFGYAVGAPERR<br>NILMVGDGSFQLTAQEVAQMVRLKLPVIIFLINNYGYTIEVMIHDGPYNNIKNWDYAGLMEVFNGNGGYDSG<br>AAKGLKAKTGGELAEAIKVALANTDGPTLIECFIGREDCTEELVKWGKRVAAANSRKPVNKVVGSTENLYFQ<br>SGALE
ZP_08570611
MSSINSFTVADYLLTRLHQLGLRKVFQVPGDYVANFMDALEQFNGIEAVGDLTELGAGYAADGYARLTGIGA<br>VSVQFGVGTFSVLNAIAGSYVERNPVVVITASPSTGNRKTIKETGVLFHHSTGDLLADSKVFANVTVAAEVLSD<br>PSDARQKIDKALTLAITFRRPIYLEAWQDVWGLACEKPEGELKALPLISEEGALKAMLADSLKLLNSARQPLV<br>LLGVEINRFGLQDAVLDLLKASGLPYSTTSLAKTVISENEGIFVGTYADGASFPATVEYIEKADCVLALGVIFTD<br>DYLTMLSKQFDQMIVVNNDETSRLGHAYYHQLYLADFILQLTDEIKKSSLYPRQNSALPLLPPQPQITPALLQ<br>QQLSYQNFFDLFYGYLLQHQLQDNISLILGESSSLYMSARLYGLPQDSFIADAAWGSLGHETGCVTGIAYASDK<br>RAMAIAGDGGFMMMCQCLSTISRHQLNSVVFVISNKVYAIEQSFVDICAFAKGGHFAPFDLLPTWDYLSLAKA<br>FSVEGYRVQNGEELLQALEHIMTQKDKPALVEVVIQSQDLAPAMAGLVKSITGHTVEQCAIPTGSTENLYFQS<br>GALE
YP_831380
MTTVHAAAYELLRSNRLTTIFGNPGDNELPFLDAMPADFRYILGLHEGVVVGMADGFAQASGQAAFVNLHA<br>ASGTGNAMGALTNAWYSHTPLVITAGQQVRPMIGLEAMLSNVDAASLPRPLVKWSAEPAQAPDVPRALSQA<br>IHTATSDPKGPVYLSIPYDDWNQDTGNLSEHLSSRSVSRAGNPSAEQLDDILSALREAANPALVFGPDVDAAR<br>ANHHAVRLAEKLAAPVWIAPAAPRCPFPTRHPNFRGVLPASIAGISALLNGHDLIVVIGAPVFRYHQYQPGSY<br>LPENSRLIHITCDAGEAARAPMGDALVADIGQTLRALADIIPQSKRPPLRPRVIPPVPDSQDDLLAPDAVFEVM<br>NEVAPEDVVYVNESVSTVTALWERVELKHPGSYYFPASGGLGFGMPAAVGVQLANDRRRVIAVIGDGSANYG<br>ITALWTAAQEKIPVVFIILNNGTYGALRAFAKLLNAENAAGLDVPGICFCAIAEGYGVEAHRITSLENFKDKLS<br>AALQSDTPTLLEVPTSTTSPFGSTENLYFQSGALE
ZP_06547677
MKTIHSAAYALLRRHGMTTIFGNPGSNELPFLKSFPEDFQYVLGLHEGAVVGMADGYALASGKPAFVNLHAA<br>AGTGNGMGALTNSWYSHSPLVITAGQQVRPMIGVEAMLANVDATQLPKPLVKWSYEPANAQDVPRALSQAI

HYANTTPKAPVYLSIPYDDWDQPSGPGVEHLIERDVQTAGTPDARQLQVLVQQVQDARNPVLVLGPDVDAT<br>LSNDHAVALADKLRMPVWIAPAASRCPFPTRHPSFRGVLPAAIAGISKTLQGHDLIIVVGAPVFRYLQFAPGD<br>YLPVGAQLLHITSDPLEATRAPMGHALVGDIRETLRVLAEEVVQQSRPYPEALAAPECVTDEPHHLHPETLFD<br>VLDAVAPHDAIYVKESTSTVTAFWQRMNLRHPGSYYFPAAGGLGFGLPAAVGVQLAQPQRRVVALIGDGSAN<br>YGITALWTAAQYRIPVVFIILKNGTYGALRWFAGVLKAEDSPGLDVPGLDFCAIAKGYGVKAVHTDTRDSFEA<br>ALRTALDANEPTVIEVPTLTIQPHGSTENLYFQSGALE
1OVM_P23234
MRTPYCVADYLLDRLTDCGADHLFGVPGDYNLQFLDHVIDSPDICWVGCANELNASYAADGYARCKGFAALL<br>TTFGVGELSAMNGIAGSYAEHVPVLHIVGAPGTAAQQRGELLHHTLGDGEFRHFYHMSEPITVAQAVLTEQN<br>ACYEIDRVLTTMLRERRPGYLMLPADVAKKAATPPVNALTHKQAHADSACLKAFRDAAENKLAMSKRTALL<br>ADFLVLRHGLKHALQKWVKEVPMAHATMLMGKGIFDERQAGFYGTYSGSASTGAVKEAIEGADTVLCVGTR<br>FTDTLTAGFTHQLTPAQTIEVQPHAARVGDVWFTGIPMNQAIETLVELCKQHVHAGLMSSSSGAIPFPQPDG<br>SLTQENFWRTLQTFIRPGDIILADQGTSAFGAIDLRLPADVNFIVQPLWGSIGYTLAAAFGAQTACPNRRVIVL<br>TGDGAAQLTIQELGSMLRDKQHPIILVLNNEGYTVERAIHGAEQRYNDIALWNWTHIPQALSLDPQSECWRV<br>SEAEQLADVLEKVAHHERLSLIEVMLPKADIPPLLGALTKALEACNNAGSTENLYFQSGALE
ZP_06418208
MGAHYPMSGESTVHDVTYQLLRSLGITTVFGNPGSTEQTFLQDFPSDFTYVLGLQEASVMAMADAFAQVTR<br>RPALVNLHSSAGVGHSIGNLVSAFDAHTPLIVTAGQQHREMVIGEPALSNREATNLPRPWVKWSYEPARAQD<br>VPEAFMRACAIATQPPAGPVFLSIPLDDWNAPMTGPAVVRSVSTVCAPETERLRGFARRIRASQRPVLVFGPE<br>VDRSGGWHAAIALAENLGVPVFGAAGPDRVSFPEDHPLFQGRLGMSQKSVSDRLTGYDLVVVIGAAVFRYYP<br>HVPGDILPAGTELLHITGDPAVAGAARVGDSVLGDARLAIELLTELLDAEAAHSPRRPQHQHPHPQPQHVEH<br>VQHQQPPQTRFQARAQPRPPRHAPDRPETAGGPLCADEVHAVINASRPRNAALVYESTSTIGEQVEWLPVIE<br>PASFFANASGGLGWAVPAAVGVALGDRDRGVHRPVIGIIGDGAFQYSVQALWTATQHSLPIVFVVLRHEYSIL<br>KSFAELERTAGVGSTENLYFQSGALE
ZP_07290467
MRTVRESALDVLRARGMTTVFGNPGSTELPMLKQFPDDFRYVLGLQEAVVVGMADGFALASGTTGLVNLHT<br>GPGTGNAMGAILNARANRTPMVVTAGQQVRAMLTMEALLTNPQSTLLPQPAVKWAYEPPRAADVAPALAR<br>AVQVAETPPQGPVFVSLPMDDFDVVLGEDEDRAAQRAAARTVTHAAAPSAEVVRRLAARLSGARSAVLVAG<br>NDVDASGAWDAVVELAERTGLPVWSAPTEGRVAFPKSHPQYRGMLPPAIAPLSRCLEGHDLVLVIGAPVFCY<br>YPYVPGAHLPENTELVHLTRDADEAARAPVGDAVVADLALTVRALLAELPAREAAAPAARTARAESTAEVD<br>GVLTPLAAMTAIAQGAPANTLWVNESPSNLGQFHDATRIDTPGSFLFTAGGGLGFGLAAAVGAQLGAPDRPV<br>VCVIGDGSTHYAVQALWTAAAYKVPVTFVVLSNQRYAILQWFAQVEGAQGAPGLDIPGLDIAAVATGYGVRA<br>HRATGFGELSKLVRESALQQDGPVLIDVPVTTELPTLGSTENLYFQSGALE
Native_KIVD
MYTVGDYLLDRLHELGIEEIFGVPGDYNLQFLDQIISRKDMKWVGNANELNASYMADGYARTKKAAAFLTTF<br>GVGELSAVNGLAGSYAENLPVVEIVGSPTSKVQNEGKFVHHTLADGDFKHFMKMHEPVTAARTLLTAENAT<br>VEIDRVLSALLKERKPVYINLPVDVAAAKAEKPSLPLKKENSTSNTSDQEILNKIQESLKNAKKPIVITGHEIISF<br>GLEKTVSQFISKTKLPITTLNFGKSSVDEALPSFLGIYNGKLSEPNLKEFVESADFILMLGVKLTDSSTGAFTHH<br>LNENKMISLNIDEGKIFNESIQNFDFESLISSLLDLSEIEYKGKYIDKKQEDFVPSNALLSQDRLWQAVENLTQS<br>NETIVAEQGTSFFGASSIFLKPKSHFIGQPLWGSIGYTFPAALGSQIADKESRHLLFIGDGSLQLTVQELGLAIRE<br>KINPICFIINNDGYTVEREIHGPNQSYNDIPMWNYSKLPESFGATEERVVSKIVRTENEFVSVMKEAQADPNR<br>MYWIELILAKEDAPKVLKKMGKLFAEQNKS
CAK95977
MKTVHGATYDILRQHGLTTIFGNPGDNELPFLKGFPEDFRYILGLHEGAVVGMADGYALASGQPTFVNLHAA<br>AGTGNGMGALTNAWYSHSPLVITAGQQVRSMIGVEAMLANVDAAQLPKPLVKWSHEPATAQDVPRALSQAI<br>HTANLPPRGPVYVSIPYDDWACEAPSGVEHLARRQVSSAGLPSPAQLQHLCERLAAARNPVLVLGPDVDGSA<br>ANGLAVQLAEKLRMPAWVAPSASRCPFPTRHACFRGVLPAAIAGISHNLAGHDLILVVGAPVFRYHQFAPGN<br>YLPAGCELLHLTCDPGEAARAPMGDALVGDIALTLEAVLDGVPQSVRQMPTALPAAEPVADDGGLLRPETVF<br>DLLNALAPKDAIYVKESTSTVGAFWRRVEMREPGSYFFPAAGGLGFGLPAAVGVQLASPGRQVIGVIGDGSAN<br>YGITALWTAAQYNIPVVFIILKNGTYGALRWFADVLDVNDAPGLDVPGLDFCAIARGYGVQAVHAATGSAFA

QALREALESDRPVLIEVPTQTIEPGSTENLYFQSGALE
ZP_07282849
MARFGVRKAFGVVGSGNFHFTNGLIQGGAEFVAARHEGGATTMADAYARCSGEVAAVSVHQGCGLGNATTG<br>IGEAAKSRTPLVVVTAEATDPLSNFHIDQDALARSVGASSILVRSAKTALADVRRAFTQARQDRRTVLLRLPL<br>EVQAEPFDESLLEGLTAIEALPQPRAAEAEVQALASILQRAERPVFLCGRGSRAARAELVALADRCGALLAEGA<br>VAKGLFAGEPWAIGVSGGFSSPLTTELIQGADVVVGWGSALNDWTTAHGRLLSPETTLVQVDLESAALGRNR<br>PVDLGIVGDVGGTALAVAELLEVHNGYRSAELKTRIAREIRWRDNEYDDVSTGEVIDPRTLSAALDELLPANR<br>VVGVDSGNFMGYPTMYLDVPDENGFCFTQAFASIGLGLATAIGTALARPDRFPVAACGDGGFLMSIAELETVV<br>RLKLPMLIVVYNDHAYGAEVYFFEPGGHPADTVTFPDTDLAAIARGYGCDAVTVRTKEDLAEVATRVAAGLD<br>RPLVVDAKIAGFSAWWLQAAMTHHGSTENLYFQSGALE
ZP_06846103
MTSRSSFSPPSASEQRGADIFAEVLQCEGVRYIFGNPGTTELPLLDALTDITGIHYVLGLHEASVVAMADGYAQ<br>ASGKPGFVNLHTAGGLGNAMGAILNAKMANTPLVVTAGQQDTRHGVTDPLLHGDLTGIARPNVKWAEEIH<br>HPEHIPMLLRRALQDCRTGPAGPVFLSLPIDTMERCTSVGAGEASRIERASVANMLHALATALAEVTAGHIAL<br>VAGEEVFTANASVEAVALAEALGAPVFGASWPGHIPFPTAHPQWQGTLPPKASDIRETLGPFDAVLILGGHSL<br>ISYPYSEGPAIPPHCRLFQLTGDGHQIGRVHETTLGLVGDLQLSLRALLPLLARKLQPQNGAVARLRQVATLKR<br>DARRTEAAERSAREFDASATTPFVAAFETIRAIGPDVPIVDEAPVTIPHVRACLDSASARQYLFTRSAILGWGM<br>PAAVGVSLGLDRSPVVCLVGDGSAMYSPQALWTAAHERLPVTFVVFNNGEYNALKNFARAQTNYRSARANR<br>FIGLDISDPAIDFPALASSLGVPARRVERAGDIAIAVEDGIRSGRPNLIDVLISSSSGSTENLYFQSGALE
ZP_04996569
MLRTAGEESGVKVRDAFFEVLRSHGITTVFGNPGSNELPLLRDFPDDFRYVLALHEGAAIAMADGYALATGR<br>PSLVNLHAAAGTGNAMGNLTNTQSGHVPVVVTSGQQARRYTALNALLTNVDATALAEPLVKWSCEPLRPED<br>VPQALSQGILLAGSAPAGPVYLSLPLDDWDHQADPGALKHLKARTVQGDPVVSEPALDLLRRRLTGAANPV<br>MVVGPGIDDATGWDGACRLADRLALPVFVAPSPSRCPFPTRHPGYRGVLPSDIPAVARHFDGHDLVVAFGAA<br>IFRYFAFEEGDYLPPGTELWAVTSDPDEATRAPFGRILVGNPSDALARLTETVPARHRPPPPPLERTSRLNEA<br>GPAFSAEAIVDALDAAKDESTVLAHEWTSVLTTWDRFDISRPGSLYFPASGGLGWGLPAAIGLQLGDPSRRVL<br>AMLGDGALHYTVSALWTAARYRVPVVFVVARNGEYGALKKFTQAMQAPGVPGLELPGIDITGIASAYGISAT<br>RIDTLDALTAAVTAALATDEPHLIEVPQQPLTASGSTENLYFQSGALE
YP_381143
MAPYRAPHRSPHSYFTMKGHEAILRQFLANGMDHMFGTPGDVEQGFLDALADVPEMKYILTLQESIAVLCA<br>DGYARARLKPALVQIHSSPGLGNAIGNLYQAMRGQAPLVVIGGDAGIKYQAMDAQMAADLVAMAEPVTKWS<br>AMVQHPSSLLRMVRRAIKVAATPPCGPVYLCLPEDILDAEITEKIIPAHIPSLETCPGSLDLDRMVSAIQSAQNP<br>IILVGDGVAWTGGVEKIVDLAETLGAKVYSADGGEINFPDDHLLNYGSTGAMFGDQSLPIMQSCDLCLTLGCY<br>LLPEVFPHLGDIFNEDATIIHVDTNVDNIAKNHRVDISYVAEPHSVVTGLLPILKSLSSSWHNAAQQRRSKLES<br>ESPVVHNNVDQNYQVEPPYPSEAYDGINRSMRSGYFIKTLADKLPKETIIFDEALTNSPPVNRYLPGQKPGDR<br>MLTRGGSLGTGFPGAIGAKIAYPDRCVIGFSGDGGSMYTIQCLWTAVRHNVAAKFIVCQNRSYKLLQSNISKF<br>WQERGIEGREFPVPFDLSKPEICFSVIANSFGVSGERVVRPDQVGEAIDRMLNHDGPYLINLVLDGDIRPDLIG<br>VRCGQGSTENLYFQSGALE

Library	Native	Mutations
1	D285<br>S286	ADLF<br>FMSTWY
2	Q377<br>F381<br>F382	AFMWQ(ILR*)[a]<br>ALF(SV) [a]<br>AFMW(IL) [a]
3	G402	AILMTV(S) [a]
4	V461<br>I465	AMWTV<br>IV
5	M538<br>F542	AHLMV(T) [a]<br>AFHIMTVWY(L) [a]

Supplementary Table 5. KIVD Mutants with Altered Specificity for Long-chain 2-Ketoacids from the Results of High-throughput Kinetic Assays. *(a) Potential mutations in parenthesis were included due to degenerate codon used for mutagenesis.

Supplementary Table 6. Partially degenerate oligonucleotides used to generate libraries of *kivd* mutants (with N-terminal histidine tags) by PCR.

**Primer Sequence**	**Description**
GAGTTAAACTCACATTNTHYTCAACAGGAGCCTTCACTCATC	primer for KIVD D285, S286 mutants D285LF S286SFY
GAGTTAAACTCACATTNAYGTCAACAGGAGCCTTCACTCATC	primer for KIVD D285, S286 mutants D285LF S286TM
GAGTTAAACTCACATTNTGGTCAACAGGAGCCTTCACTCATC	primer for KIVD D285, S286 mutants D285LF S286W
GAGTTAAACTCACAGMYTHYTCAACAGGAGCCTTCACTCATC	primer for KIVD D285, S286 mutants D285AD S286SFY
GAGTTAAACTCACAGMYAYGTCAACAGGAGCCTTCACTCATC	primer for KIVD D285, S286 mutants D285AD S286TM
GAGTTAAACTCACAGMYTGGTCAACAGGAGCCTTCACTCATC	primer for KIVD D285, S286 mutants D285AD S286W
CAATCGTTGCTGAAGCNGGGACATCAKYNGCNGGCGCTTCATCAATTTTCTTAAAAC	primer for KIVD Q377, F381, F382 mutants Q377A F381LAFVS F382A
CAATCGTTGCTGAAGCNGGGACATCAKYNWTKGGCGCTTCATCAATTTTCTTAAAAC	primer for KIVD Q377, F381, F382 mutants Q377A F381LAFVS F382MFIL
CAATCGTTGCTGAAGCNGGGACATCAKYNTGGGGCGCTTCATCAATTTTCTTAAAAC	primer for KIVD Q377, F381, F382 mutants Q377A F381LAFVS F382W
CAATCGTTGCTGAAWTKGGGACATCAKYNGCNGGCGCTTCATCAATTTTCTTAAAAC	primer for KIVD Q377, F381, F382 mutants Q377MFIL F381LAFVS F382A
CAATCGTTGCTGAAWTKGGGACATCAKYNWTKGGCGCTTCATCAATTTTCTTAAAAC	primer for KIVD Q377, F381, F382 mutants Q377MFIL F381LAFVS F382MFIL
CAATCGTTGCTGAAWTKGGGACATCAKYNTGGGGCGCTTCATCAATTTTCTTAAAAC	primer for KIVD Q377, F381, F382 mutants Q377MFIL F381LAFVS F382W
CAATCGTTGCTGAAYRGGGGACATCAKYNGCNGGCGCTTCATCAATTTTCTTAAAAC	primer for KIVD Q377, F381, F382 mutants Q377WQR* F381LAFVS F382A
CAATCGTTGCTGAAYRGGGGACATCAKYNWTKGGCGCTTCATCAATTTTCTTAAAAC	primer for KIVD Q377, F381, F382 mutants Q377WQR* F381LAFVS F382MFIL
CAATCGTTGCTGAAYRGGGGACATCAKYNTGGGGCGCTTCATCAATTTTCTTAAAAC	primer for KIVD Q377, F381, F382 mutants Q377WQR* F381LAFVS F382W
GTCAACCCTTATGGNTRTCAATTGGATATACATTCCCAGCAG	primer for KIVD G402 mutants G402VILM
GTCAACCCTTATGGRSYTCAATTGGATATACATTCCCAGCAG	primer for KIVD G402 mutants G402GATS
AATGATGGTTATACARYGGAAAGAGAARTHCATGGACCAAATCAAAGCTACAATG	primer for KIVD V461, I465 mutants V461MTAV I465IV
AATGATGGTTATACATGGGAAAGAGAARTHCATGGACCAAATCAAAGCTACAATG	primer for KIVD V461, I465 mutants V461W I465IV
AAAGTACTGAAAAAACWYGGCAAACTAYWYGCTGAACAAAATAAATCATAAGCATG	primer for KIVD M538, F542 mutants M538LH F542YHFL
AAAGTACTGAAAAAACWYGGCAAACTARYNGCTGAACAAAATAAATCATAAGCATG	primer for KIVD M538, F542 mutants M538LH F542MITAV

AAAGTACTGAAAAAACWYGGCAAACTATGGGCTGAACAAAATAAATCATAAGCATG	primer for KIVD M538, F542 mutants M538LH F542W
AAAGTACTGAAAAAARYGGGCAAACTAYWYGCTGAACAAAATAAATCATAAGCATG	primer for KIVD M538, F542 mutants M538VAMT F542YHFL
AAAGTACTGAAAAAARYGGGCAAACTARYNGCTGAACAAAATAAATCATAAGCATG	primer for KIVD M538, F542 mutants M538VAMT F542MITAV
AAAGTACTGAAAAAARYGGGCAAACTATGGGCTGAACAAAATAAATCATAAGCATG	primer for KIVD M538, F542 mutants M538VAMT F542W
GTCAACCCTTATGGTCTTCAATTGGATATACATTCCCAGCAG	KIVD G402S mutation primer
GTCAACCCTTATGGGTGTCAATTGGATATACATTCCCAGCAG	KIVD G402V mutation primer
AATGATGGTTATACATGGGAAAGAGAAATTCATGGACCAAATCAAAGC	KIVD V461W mutation primer
AATGATGGTTATACAGCGGAAAGAGAAGTTCATGGACCAAATCAAAGCTACAATG	KIVD V461A I465V mutation primer
AATGATGGTTATACATGGGAAAGAGAAGTTCATGGACCAAATCAAAGCTACAATG	KIVD V461W I465V mutation primer
AATGATGGTTATACAATGGAAAGAGAAGTTCATGGACCAAATCAAAGCTACAATG	KIVD V461M I465V mutation primer
AATGATGGTTATACAATGGAAAGAGAAATTCATGGACCAAATCAAAGC	KIVD V461M mutation primer
AAAGTACTGAAAAAAcatGGCAAACTActgGCTGAACAAAATAAATCATAAGCATG	primer for KIVD M538, F542 mutants M538H F542L
AAAGTACTGAAAAAAatgGGCAAACTAgtgGCTGAACAAAATAAATCATAAGCATG	primer for KIVD M538, F542 mutant F542V
AAAGTACTGAAAAAAcatGGCAAACTAtttGCTGAACAAAATAAATCATAAGCATG	primer for KIVD M538, F542 mutants M538H
AAAGTACTGAAAAAActgGGCAAACTAaccGCTGAACAAAATAAATCATAAGCATG	primer for KIVD M538, F542 mutants M538L F542T
AAAGTACTGAAAAAAcatGGCAAACTAtggGCTGAACAAAATAAATCATAAGCATG	primer for KIVD M538, F542 mutants M538H F542W
AAAGTACTGAAAAAAcatGGCAAACTAaccGCTGAACAAAATAAATCATAAGCATG	primer for KIVD M538, F542 mutants M538H F542T
AAAGTACTGAAAAAActgGGCAAACTAgtgGCTGAACAAAATAAATCATAAGCATG	primer for KIVD M538, F542 mutants M538L F542V
AAAGTACTGAAAAAAcatGGCAAACTAgcgGCTGAACAAAATAAATCATAAGCATG	primer for KIVD M538, F542 mutants M538H F542A
GAGTTAAACTCACACTGTTTTCAACAGGAGCCTTCACTCATC	primer for KIVD mutant D285L S286F
GAGTTAAACTCACAGACTGGTCAACAGGAGCCTTCACTCATC	primer for KIVD mutant S286W
CAATCGTTGCTGAAATGGGGACATCATTCCTGGGCGCTTCATCAATTTTCTTAAAAC	primer for KIVD mutant Q377M F382L
CAATCGTTGCTGAAATTGGGACATCACTGTGGGGCGCTTCATCAATTTTCTTAAAAC	primer for KIVD mutant Q377I F381L F382W
AAAGTACTGAAAAAAcatGGCAAACTAatgGCTGAACAAAATAAATCATAAGCATG	primer for KIVD M538, F542 mutants M538H F542M
AAAGTACTGAAAAAAcatGGCAAACTAcatGCTGAACAAAATAAATCATAAGCATG	primer for KIVD M538, F542 mutants M538H F542H
AAAGTACTGAAAAAAgtgGGCAAACTAgtgGCTGAACAAAATAAATCATAAGCATG	primer for KIVD M538, F542 mutants M538V F542V
TCCGACTTACTCTGAAGACGCGTCTGGCATCACCGCGAAATAATCAAATG<br>GTGCCAGTATGAAGACTCCGTAAACGTTTCCCCCGCGAGTCAAATGTATG	Left primer for rhtC knockout<br>Right primer for rhtC knockout
ATGCGACCCTTGTGTATCAAACAAGACGATTAAAAATCTTCGTTAGTTTC	Left primer for recA knockout
CAGAACATATTGACTATCCGGTATTACCCGGCATGACAGGAGTAAAAATG	Right primer for recA knockout

CCGTTTATGTTGCCAGACAGCGCTACTGATTAAGCGGATTTTTTCGCTTT	Left primer for adhE knockout
CGAGCAGATGATTTACTAAAAAAGTTTAACATTATCAGGAGAGCATTATG	Right primer for adhE knockout

Supplementary Table 7. All *E. coli* strains and plasmids used in this study.

**Name**	**Relevant Genotype**	**Source**
Strains		
XL-1 Blue	*recA1 endA1 gyrA96 thi-1 hsdR17 supE44 relA1 lac* [F´ *proAB lac*Iq*ZΔM15* Tn*10* (Tetr)].	Stratagene, La Jolla, California
KS145	$rrnB_{T14}$ *ΔlacZWJ16 hsdR514* $\Delta araBAD_{AH33}$ $\Delta rhaBAD_{LD78}$ *ΔilvB ΔilvI* [F´ *proAB lacIqZΔM15* Tn*10* (Tetr)].	(Atsumi et. al. 2008a)
ATCC98082 *ΔrhtA*	$ilvA_{442}$ *supE spoT* $thrC_{1010}$ *Sac+ ThrR ΔrhtA*	Zhang et. al. 2010
EcRJM191	ATCC 98082 *ΔrhtA ΔrecA*	This study
ST128	ATCC 98082 *ΔrhtA ΔadhE ΔrecA*	This study
ST139	ATCC 98082 *ΔrhtA ΔadhE ΔrecA* pZS_thrO, pZAlac_ilvA_{BS}leuA, pZE_LeuA*BCDKA6_kivd_wt	This study
ST140	ATCC 98082 *ΔrhtA ΔadhE ΔrecA* pZS_thrO, pZAlac_ilvA_{BS}leuA, pZE_LeuA*BCDKA6_kivd_G402V/M538L/F542V	This study
ST141	ATCC 98082 *ΔrhtA ΔadhE ΔrecA* pZS_thrO, pZAlac_ilvA_{BS}leuA, pZE_LeuA*BCDKA6_kivd_F381L/V461A	This study
Plasmids		
pQE9	colE1, *bla*, $P_{T5/lac}$ MCS	Qiagen
pQE_hiskivd_wt	colE1, *bla*, $P_{T5/lac}$ his_6kivd	Zhang et. al. 2008
pZS_thrO	pSC101, *aadA*, P_{LlacO1} $thrA^{fbr}BC$	Zhang et. al. 2008
pZE_LeuA*BCDKA6	colE1, *bla*, P_{LlacO1} *leuA*BCD kivd adh6*	Marcheschi et. al. 2012
pZAlac_ilvA_{BS}leuA	p15A, *aphA*, $lacI^q$, P_{LlacO1} $ilvA_{BS}$ $leuA^{fbr}$	Marcheschi et. al. 2012
